# Supplementary material for: Synthesis and Characterisation of Platinum(II) Diaminocyclohexane Complexes with Pyridine Derivatives as Anticancer Agents
Source: Int J Mol Sci. 2023 Dec 5;24(24):17150. doi: 10.3390/ijms242417150 (PMC10742472; doi:10.3390/ijms242417150)
Supplement: Supplementary file 1 [file ijms-24-17150-s001.zip › ijms-2738569-supplementary.pdf]

Synthesis and Characterisation of Platinum(II) Diaminocyclohexane Complexes with Pyridine Derivatives as Anticancer Agents

Brondwyn S. McGhie,[a] Jennette Sakoff,[b] Jayne Gilbert,[b] Christopher P. Gordon[a] and Janice R. Aldrich-Wright[a*]

[a] *School of Science, Nanoscale Organisation and Dynamics Group, Western Sydney University, Locked Bag 1797 Penrith South DC, 2751, NSW, Australia*

[b] *Calvary Mater Newcastle, Waratah, NSW 2298, Australia*

**Corresponding author – Email. J.Aldrich-Wright@westernsydney.edu.au, Ph. +61246203218*

Supplementary Data

Synthesis and Characterisation of Pt(II) Diaminocyclohexane Complexes with Pyridine Derivatives as anticancer agents 1

Characterisation 3

NMR 3

[Pt(PyPy)(Cl)$_2$] 5

[Pt(ImPy)(Cl)$_2$] 6

[Pt(BImPy)(Cl)$_2$] 8

[Pt(PyPy)(*SS*DACH)]$^{2+}$ 9

[Pt(PyPy)(*RR*DACH)]$^{2+}$ 11

[Pt(ImPy)(*SS*DACH)]$^{2+}$ 12

[Pt(ImPy)(*RR*DACH)]$^{2+}$ 14

[Pt(BImPy)(*SS*DACH)]$^{2+}$ 15

[Pt(BImPy)(*RR*DACH)]$^{2+}$ 17

Molar Absorption Coefficients 19

[Pt(PyPy)(*SS*DACH)]$^{2+}$ 19

[Pt(PyPy)(*RR*DACH)]$^{2+}$ 19

[Pt(ImPy)(*SS*DACH)]$^{2+}$ 20

[Pt(ImPy)(*RR*DACH)]$^{2+}$ 20

[Pt(BImPy)(*SS*DACH)]$^{2+}$ 21

[Pt(BImPy)(*RR*DACH)]$^{2+}$ 21

HPLC 22

[Pt(PyPy)(*SS*DACH)]$^{2+}$ 22

[Pt(ImPy)(*SS*DACH)]$^{2+}$ 22

[Pt(BImPy)(*SS*DACH)]$^{2+}$ 22

[Pt(PyPy)(*RR*DACH)]$^{2+}$ 23

[Pt(ImPy)(*RR*DACH)]$^{2+}$ 23

[Pt(BImPy)(*RR*DACH)]$^{2+}$ 23
CD 24
[Pt(PyPy)(*SS*DACH)]$^{2+}$ 24
[Pt(ImPy)(*SS*DACH)]$^{2+}$ 25
[Pt(BImPy)(*SS*DACH)]$^{2+}$ 26
ESI MS 27
[Pt(PyPy)(*SS*DACH)]$^{2+}$ 27
[Pt(PyPy)(*RR*DACH)]$^{2+}$ 28
[Pt(ImPy)(*SS*DACH)]$^{2+}$ 29
[Pt(ImPy)(*RR*DACH)]$^{2+}$ 30
[Pt(BImPy)(*SS*DACH)]$^{2+}$ 31
[Pt(BImPy)(*RR*DACH)]$^{2+}$ 32
Lipophilicity 33
GI_{50} 34
References 35

Characterisation

NMR

NMR Spectral data were obtained using a 400 M*Hz* Bruker Avance spectrometer at 298 K, using 10 mm samples prepared in DMSO. ^{1}H NMR spectra were obtained using a spectral width of 8250 *Hz* and 65536 data points, while ^{195}Pt NMR spectra were acquired using a spectral width of 85470 *Hz* and 674 data points. ^{1}H-^{195}Pt HMQC spectra were recorded using a spectral width of 214436 *Hz* and 256 data points for the ^{195}Pt nucleus (F1 dimension) and a spectral width of 4808 *Hz* with 2048 data points for the ^{1}H nucleus (F2 dimension). Chemical shifts are reported in parts per million (ppm) with J coupling reported in *Hz*.

To assign each peak they were first integrated and assessed as either a doublet (d, triplet (t), doublet of doublets (dd) or a multiplet (m). Then the location and J coupling of the peaks was assessed to determine the assignment of each peak if further queries of identity arose the COSY and HMQC spectra could be utilised to distinguish between close peaks or asses which protons made up merged peaks. All proton assignments are summarised in the table below.

Table S1: Proton assignment, and J coupling for complexes **1-6** and the ^{195}Pt value.

Proton	Complex								
	**1**	**2**	**3**	**4a**	**4b**	**5a**	**5b**	**6a**	**6b**
1	δ 9.15(d, *J*= 5.85 *Hz*, H1)	δ 9.28(d, *J*= 5.74 *Hz*, H1)	δ 9.49 (d, *J*= 6.03 *Hz*, H1)	δ 9.81(d, *J*= 5.74 *Hz*, H1)	δ 8.47(d, *J*= 5.78 *Hz*, H1)	δ 8.70d, *J*= 5.73 *Hz*, H1)	δ 8.18d, *J*= 5.69*Hz*, H1)	δ 8.67(d, *J*= 5.57 *Hz*, H1)	δ 8.66(d, *J*= 5.57 *Hz*, H1)
2	δ 7.49(t, *J*= 6.88 *Hz*, H1)	δ 7.81(m, H1)	δ 7.80(merged with 12, H2)	δ 8.69(t, *J*= 6.76 *Hz*, H1)	δ 7.36(t, *J*= 6.91 *Hz*, H1)	δ 7.64(t, *J*= 6.54 *Hz*, H1)	δ 7.28(t, *J*= 6.63 *Hz*, H1)	δ 7.18(m, H1)	δ 7.19(m, H1)
3	δ 8.16(t, *J*= 7.78 *Hz*, H1)	δ 8.34(t, *J*= 7.75 *Hz*, H1)	δ 8.45(t, *J*= 7.58 *Hz*, H1)	δ 9.45(t, *J*= 7.70 *Hz*, H1)	δ 8.12(t, *J*= 7.76 *Hz*, H1)	δ 8.35(t, *J*= 7.67 *Hz*, H1)	δ 8.04(t, *J*= 7.93 *Hz*, H1)	δ 8.35(t, *J*= 7.88 *Hz*, H1)	δ 8.32(t, *J*= 7.78 *Hz*, H1)
4	δ 7.59 (d, *J*= 7.93 *Hz*, H1)	δ 8.15 (d, *J*= 8.15 *Hz*, H1)	δ 8.81 (d, *J*= 8.22 *Hz*, H1)	δ 9.14(d, *J*= 8.07 *Hz*, H1)	δ 7.81d, *J*= 8.13 *Hz*, H1)	δ 7.40(d, *J*= 7.90 *Hz*, H1)	δ 7.76(d, *J*= 7.90 *Hz*, H1)	δ 8.30d, *J*= 7.39 *Hz*, H1)	δ 8.32d, *J*= 7.40 *Hz*, H1)
6	δ 4.60(dd, *J*= 15.24, 7.71 *Hz*, H1)			δ 8.67(merged, *J* = 10.17 *Hz*, H3)	δ 7.13(merged, *J* = 9.88 *Hz*, H3)				
7	δ 2.47 (m, H merged with solvent peak)								
8	δ 1.86 (m, H2)	δ 7.37 (m, H2)		δ 7.44, 7.30(t, *J*= 9.46 *Hz*, H2)	δ 6.12, 5.96(t, *J*= 10.41 *Hz*, H2)	δ 6.03 (d, *J*= 10.20 *Hz*, H1)	δ 7.25 (d, *J*= 0.40 *Hz*, H2=1)		
9	δ 3.09 (m, H2)	δ 7.68 (m, H2)	δ 8.31 (d, *J*= 7.73 *Hz*, H1)	δ 8.14, 8.03 (d, *J*= 8.45 *Hz*, H2)	δ 6.81, 6.69 (d, *J*= 7.48 *Hz*, H2)	δ 7.15, 7.05 (d, *J*= 7.65 *Hz*, H1)	δ 7.03(d, *J*= 0.25 *Hz*, H1)	δ 7.37 (d, *J*= 7.98 *Hz*, H1)	δ 7.37 (d, *J*= 7.92 *Hz*, H1)
10			δ 7.44(t, *J*= 7.85 *Hz*, H1)					δ 7.08, 6.90(m, H2)	δ 7.08, 6.82(m, H1)
11			δ 7.52(t, *J*= 7.19 *Hz*, H1)					δ 6.61, 6.11 (m, H1)	δ 6.61, 6.11 (m, H1)
12			δ 7.80(merged with 2, H2)					δ 7.65 (d, *J*= 7.83 *Hz*, H1)	δ 7.65 (d, *J*= 7.68 *Hz*, H1)
1’,2’				δ 4.26 (m, H2)	δ 2.93 (m, H2)	δ 3.20 (m, merged with solvent peak)	δ 2.95 (m, H2)	δ 2.40 (m, merged with solvent peak)	δ 2.42 (m, merged with solvent peak)
1’,2’				δ 3.71 (m, H2)	δ 2.36 (m, H2)	δ 2.40 (m, merged with solvent peak)	δ 2.49 (m, H2)	δ 2.12 (m, H2)	δ 2.11 (m, H2)
3’,6’				δ 3.56 (m, H2)	δ 2.02 (m, H2)	δ 2.06 (m, H2)	δ 2.05 (m, H2)	δ 2.02 (m, H2)	δ 2.02 (m, H2)
3’,6’				δ 2.72 (m, H2)	δ 1.38(m, H2)	δ 1.43(m, H2)	δ 1.34(m, H4 merged with 3’/6’)	δ 1.46 (m, H2)	δ 1.46 (m, H2)
4’,5’				δ 2.96 (m, H2)	δ 1.62 (m, H2)	δ 1.58 (m, H2)	δ 1.64 (m, H2)	δ 1.60 (m, H2)	δ 1.61 (m, H2)
4’,5’				δ 2.49(m, H2)	δ 1.16(m, H2)	δ 1.15(m, H2)	δ 1.18(m, H4 merged with 3’/6’)	δ 1.18 (m, H2)	δ 1.18 (m, H2)
^{195}Pt	-3115.8	-3150.1	-2217.4	-2898.1	-2900.8	-2896.0	-2900.8	-2848.1	-2850.1

$[Pt(PyPy)(Cl)_2]$

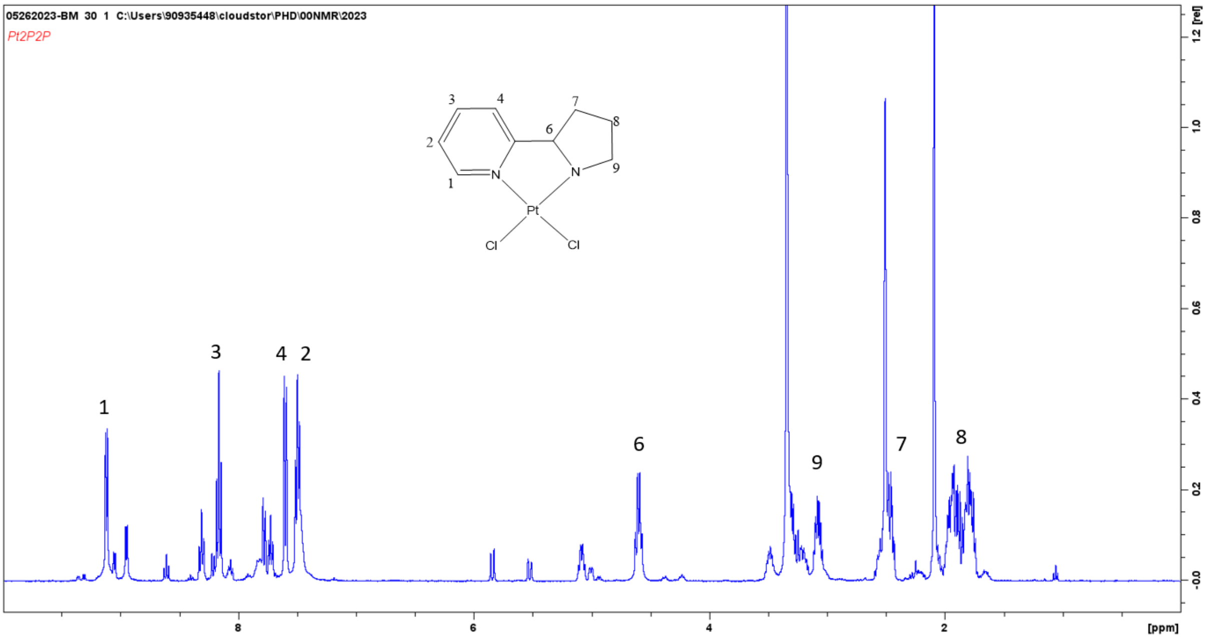

Figure S1: ^{1}H NMR spectra of $1[Pt(PyPy)(Cl)_2]^{2+}$ in DMSO, spectral width of 8250 Hz and 65536 data points.

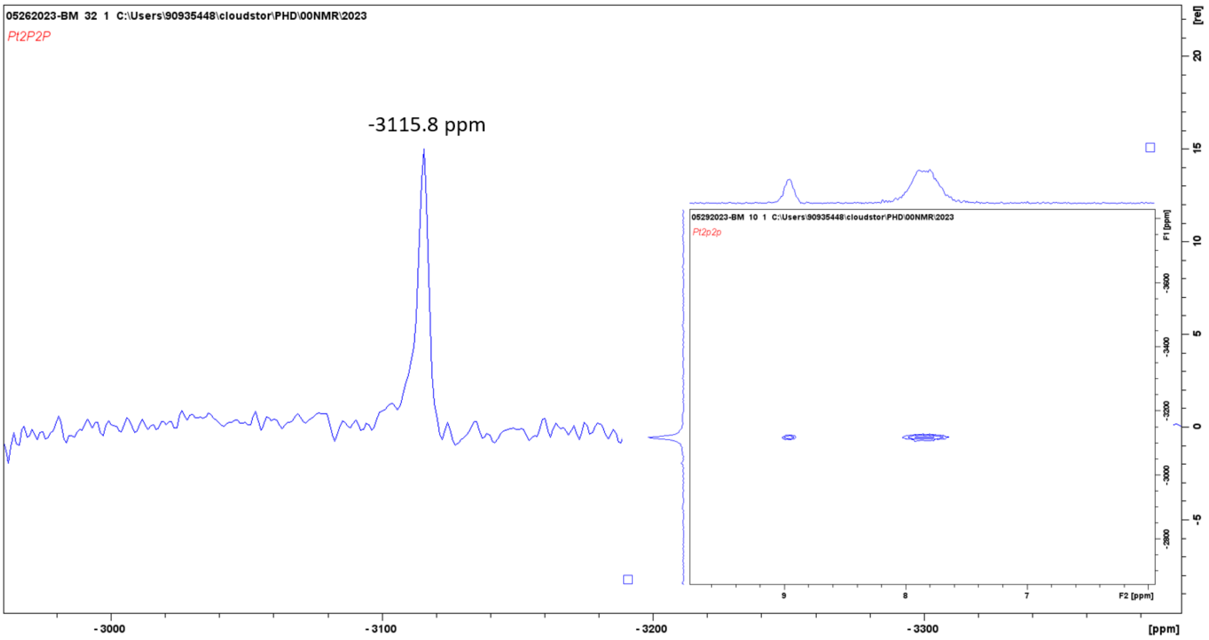

Figure S2: ^{195}Pt peak spectra of 1 $[Pt(PyPy)(Cl)_2]^{2+}$ in DMSO, spectral width of 214436 Hz and 256 data points for the ^{195}Pt nucleus (F1 dimension) and a spectral width of 4808 Hz with 2048 data points for the ^{1}H nucleus (F2 dimension).

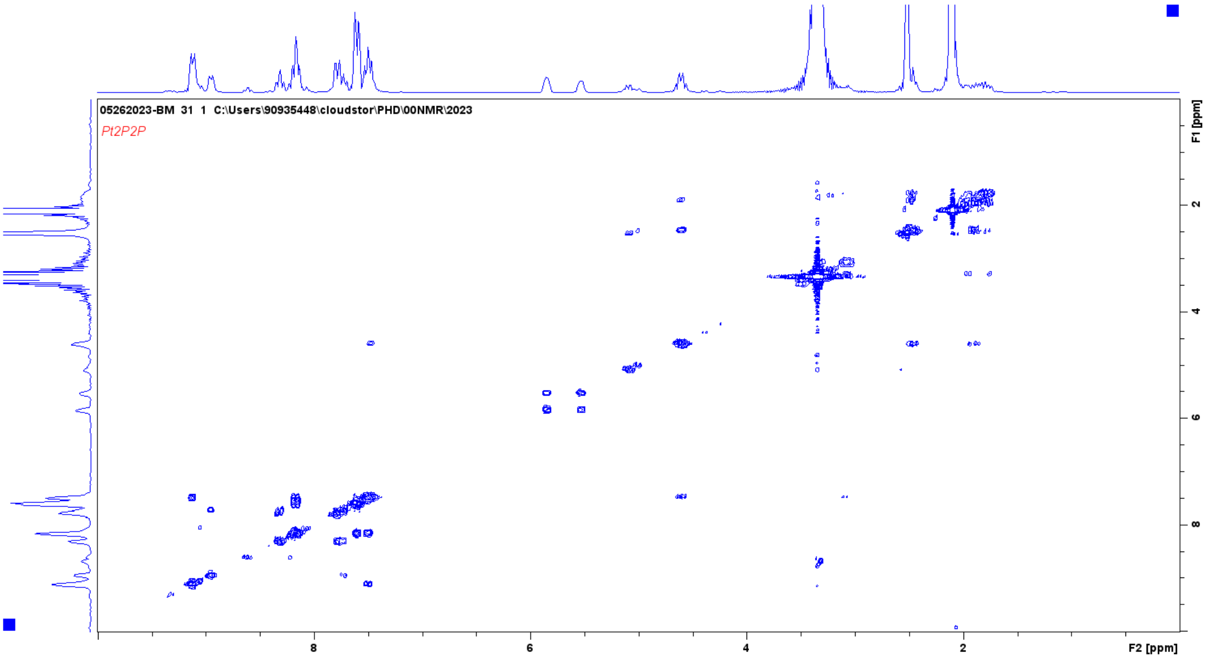

Figure S3: ^{1}H/ ^{1}H COSY spectra of 1 $[Pt(PyPy)(Cl)_2]^{2+}$ in DMSO, F1/F2 ^{1}H dimension spectral width of 8250 Hz and 65536 data points

[Pt(ImPy)(Cl)$_2$]

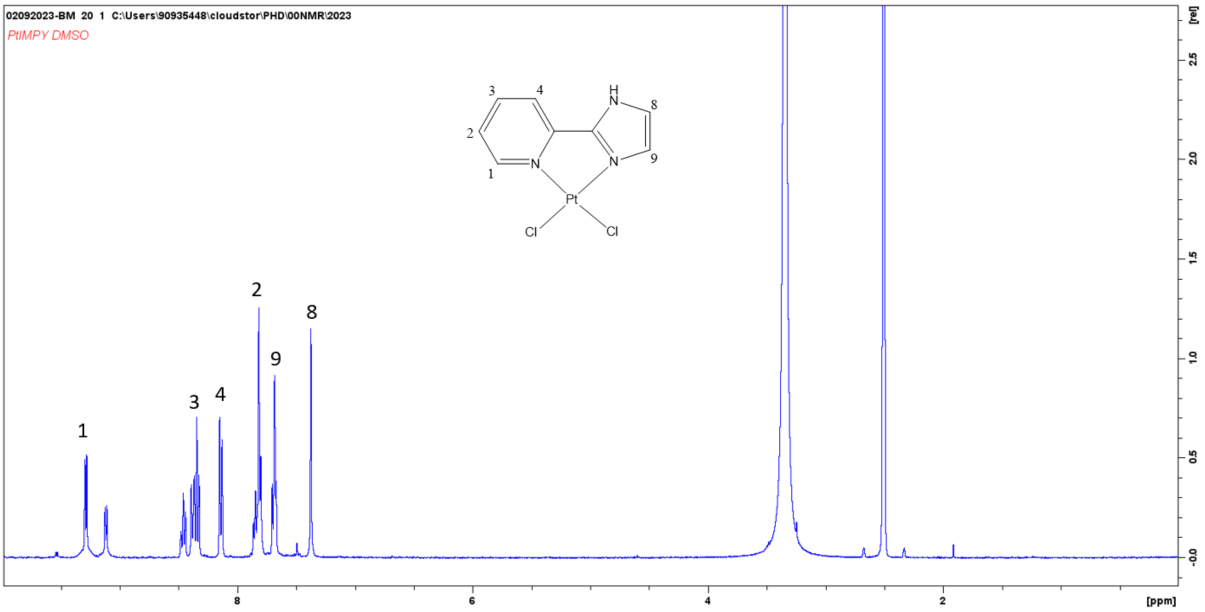

Figure S4: ^{1}H NMR spectra of 2 $[Pt(ImPy)(Cl)_2]^{2+}$ in DMSO, spectral width of 8250 Hz and 65536 data points.

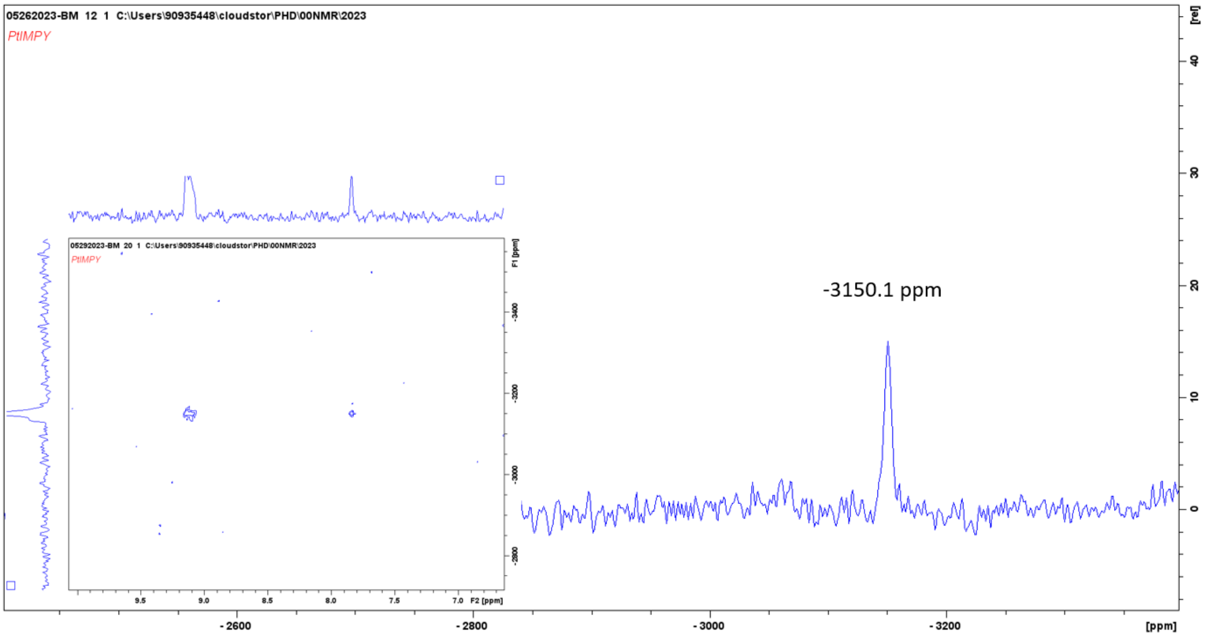

Figure S5: ^{195}Pt peak spectra of 2 $[Pt(ImPy)(Cl)_2]^{2+}$ in DMSO, spectral width of 214436 Hz and 256 data points for the ^{195}Pt nucleus (F1 dimension) and a spectral width of 4808 Hz with 2048 data points for the ^{1}H nucleus (F2 dimension).

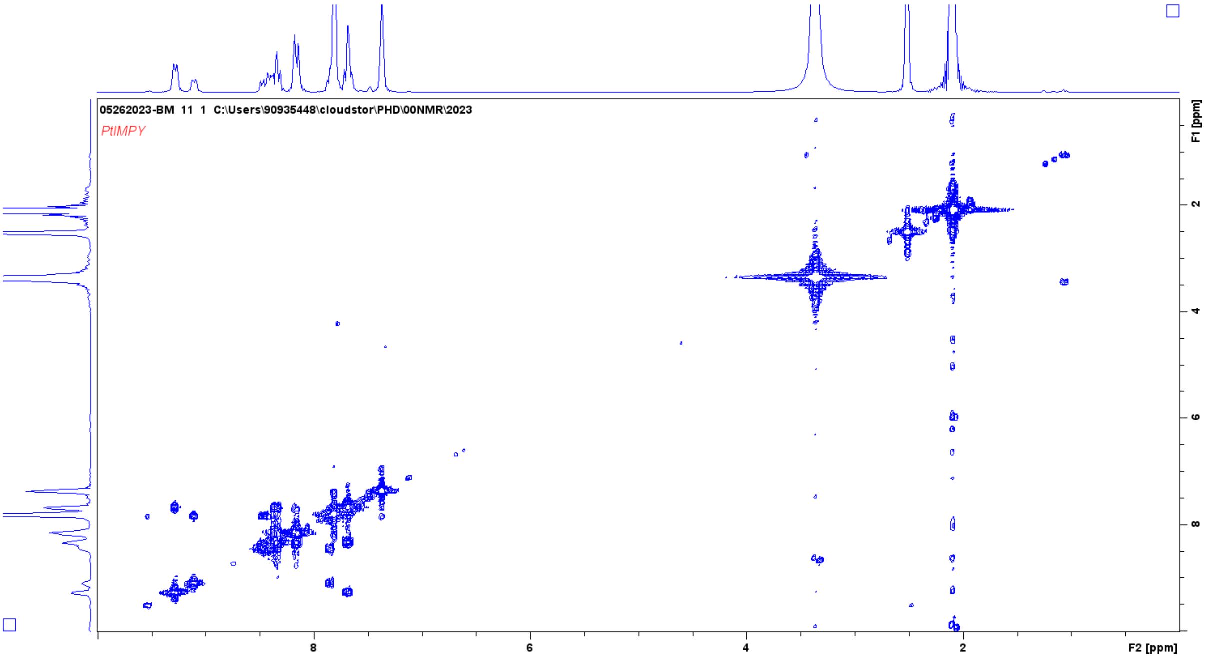

Figure S6: ^{1}H/ ^{1}H COSY spectra of 2 $[Pt(ImPy)(Cl)_2]^{2+}$ in DMSO, F1/F2 ^{1}H dimension spectral width of 8250 Hz and 65536 data points.

[Pt(BImPy)(Cl)2]

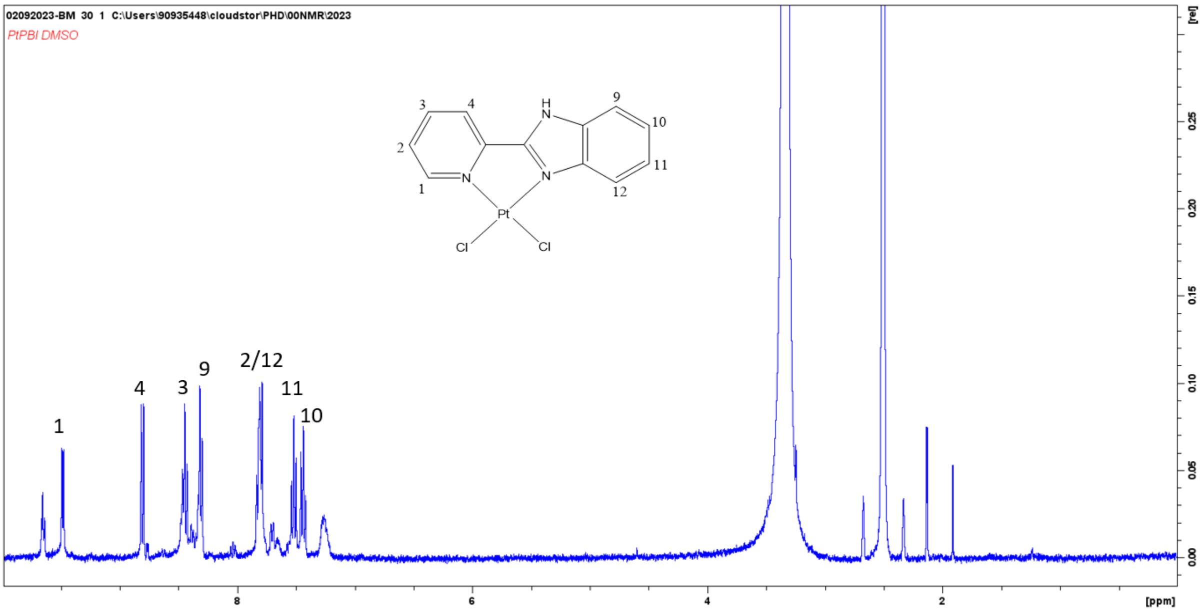

Figure S7: ^{1}H NMR spectra of 3 $[Pt(BImPy)(Cl)_2]^{2+}$ in DMSO, spectral width of 8250 Hz and 65536 data points.

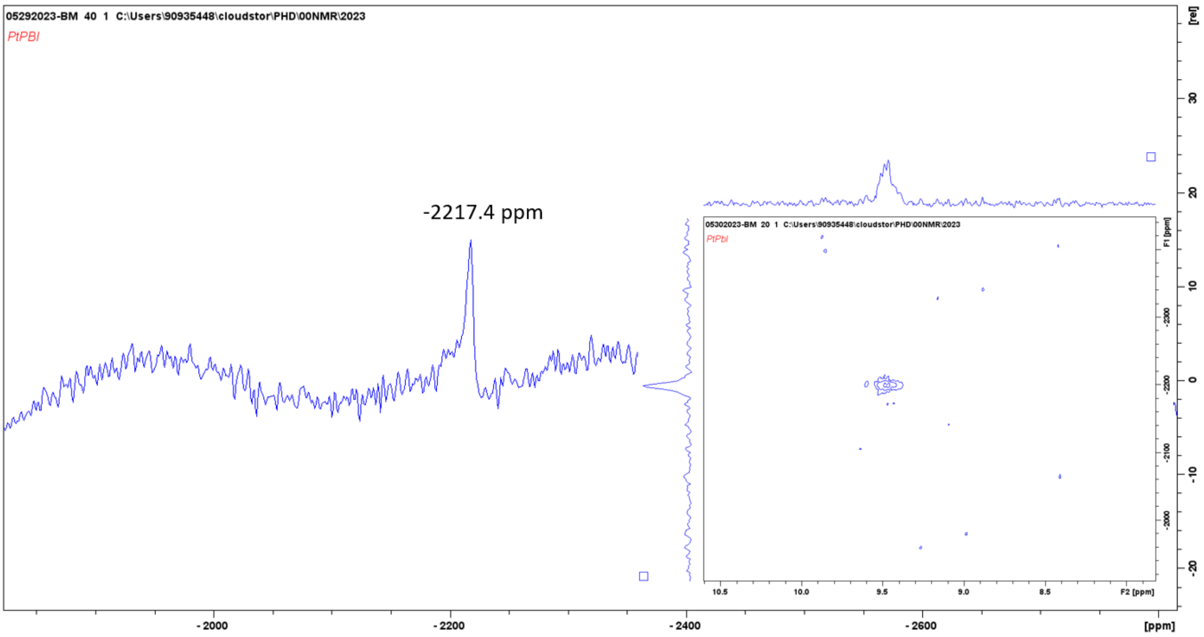

Figure S8: ^{195}Pt peak spectra of 3 $[Pt(BImPy)(Cl)_2]^{2+}$ in DMSO, spectral width of 214436 Hz and 256 data points for the ^{195}Pt nucleus (F1 dimension) and a spectral width of 4808 Hz with 2048 data points for the ^{1}H nucleus (F2 dimension).

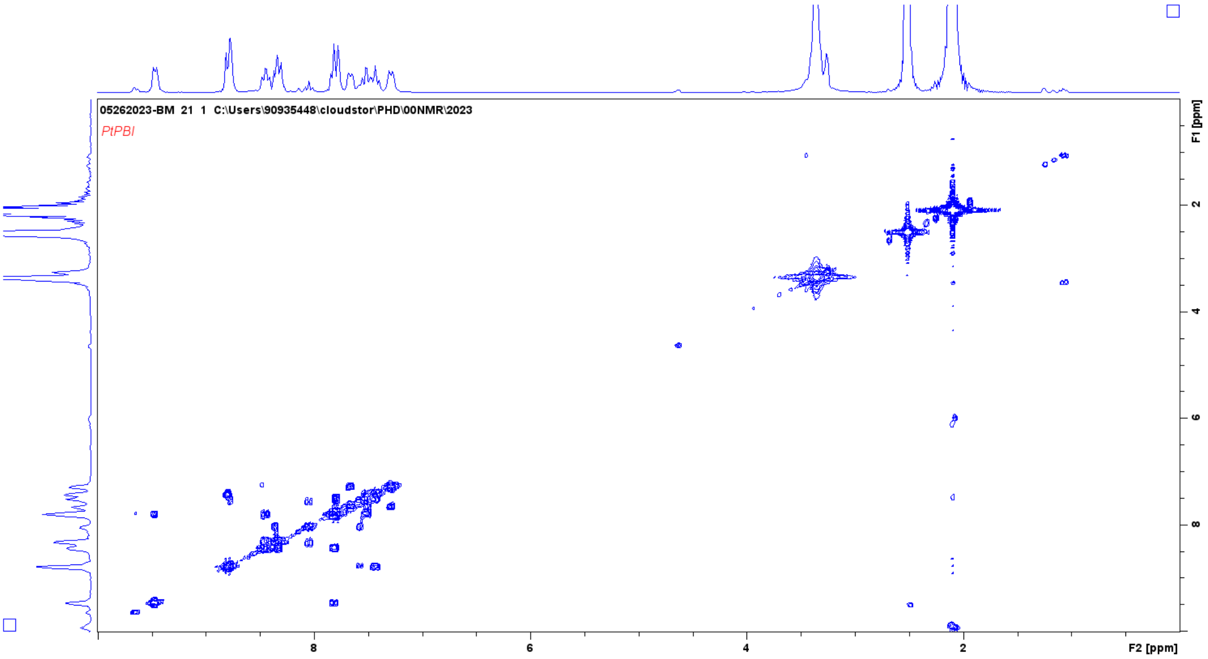

Figure S9: $^{1}H/ ^{1}H$ COSY spectra of 3 $[Pt(BImPy)(Cl)_2]^{2+}$ in DMSO, F1/F2 ^{1}H dimension spectral width of 8250 Hz and 65536 data points.

$[Pt(PyPy)(\textit{SS}DACH)]^{2+}$

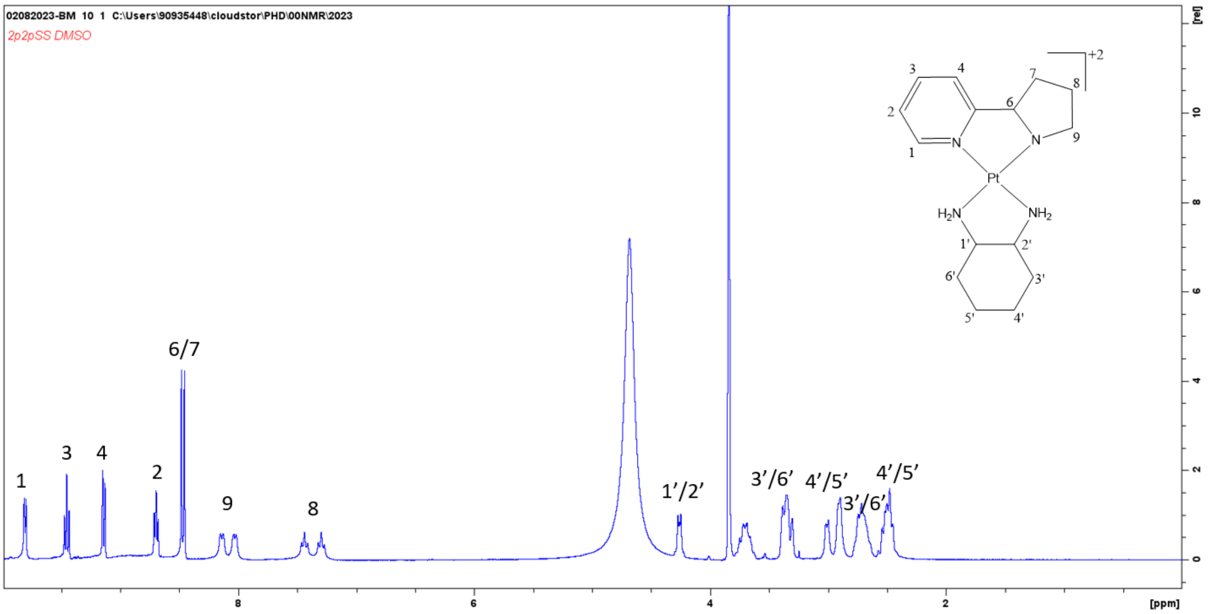

Figure S10: ^{1}H NMR spectra of 4a $[Pt(PyPy)(\textit{SS}DACH)]^{2+}$ in DMSO, spectral width of 8250 Hz and 65536 data points.

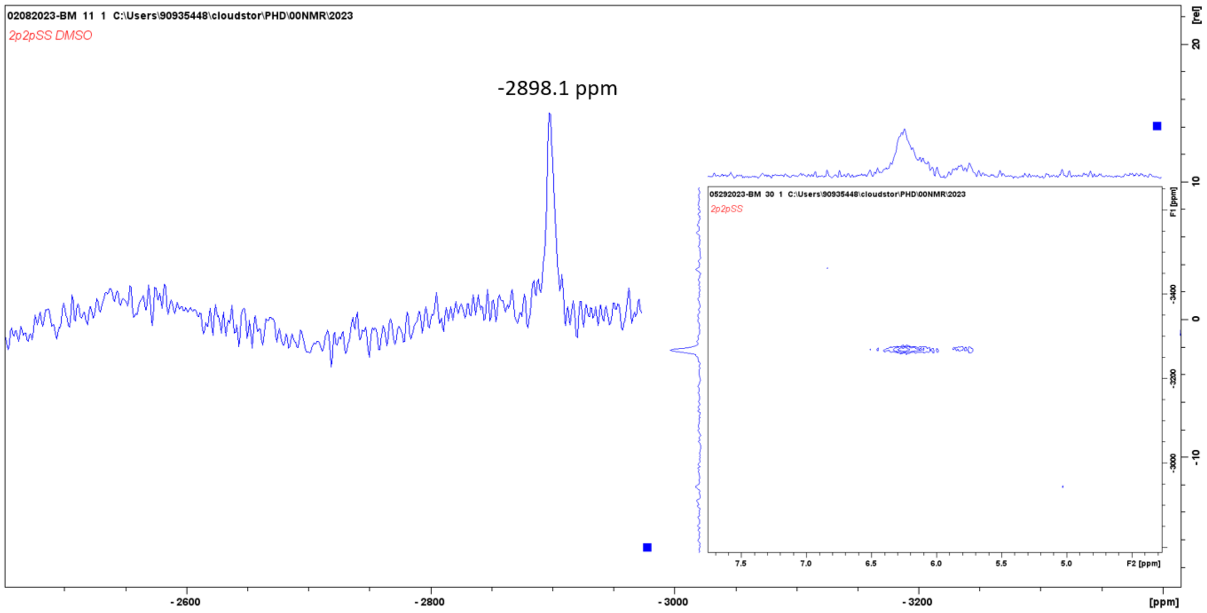

Figure S11: ^{195}Pt peak spectra of 4a [Pt(PyPy)(*SS*DACH)]$^{2+}$ in DMSO, spectral width of 214436 Hz and 256 data points for the ^{195}Pt nucleus (F1 dimension) and a spectral width of 4808 Hz with 2048 data points for the ^{1}H nucleus (F2 dimension).

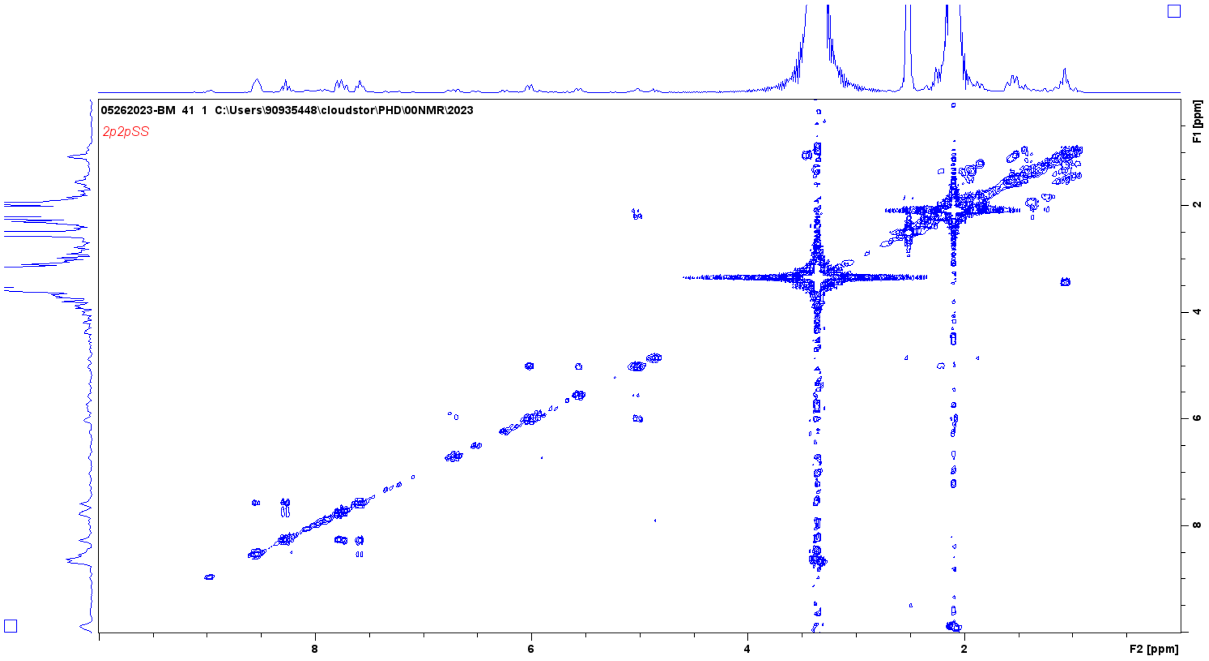

Figure S12: ^{1}H/ ^{1}H COSY spectra of 4a [Pt(PyPy)(*SS*DACH)]$^{2+}$ in DMSO, F1/F2 ^{1}H dimension spectral width of 8250 Hz and 65536 data points.

[Pt(PyPy)(*RR*DACH)]$^{2+}$

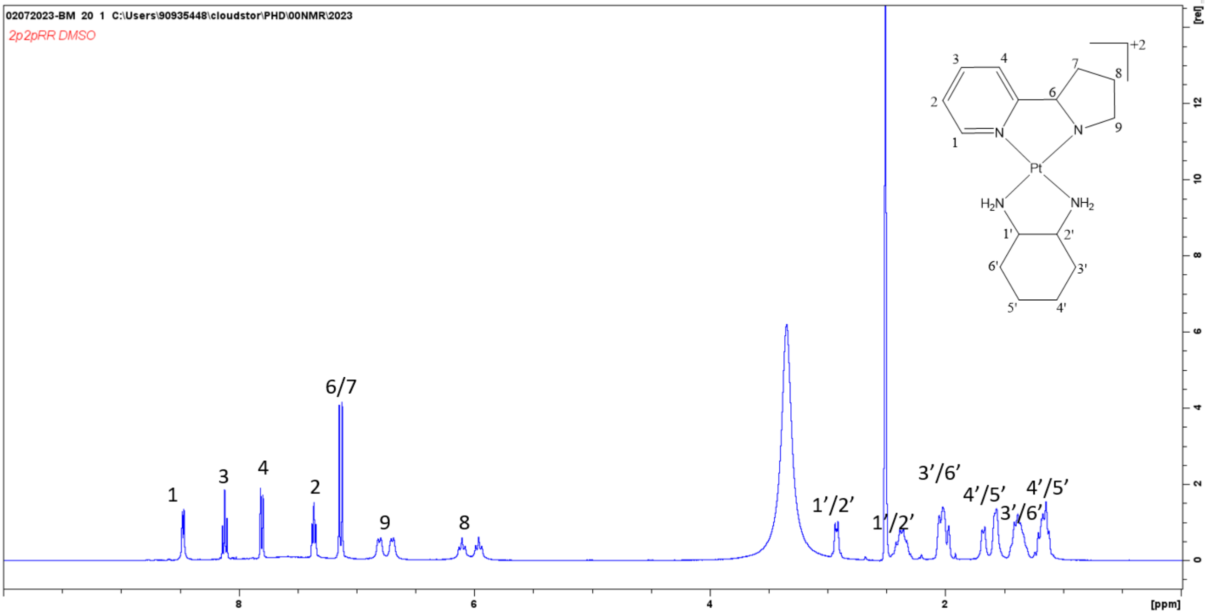

Figure S13: ^{1}H NMR spectra of 4b [Pt(PyPy)(*RR*DACH)]$^{2+}$ in DMSO, spectral width of 8250 Hz and 65536 data points.

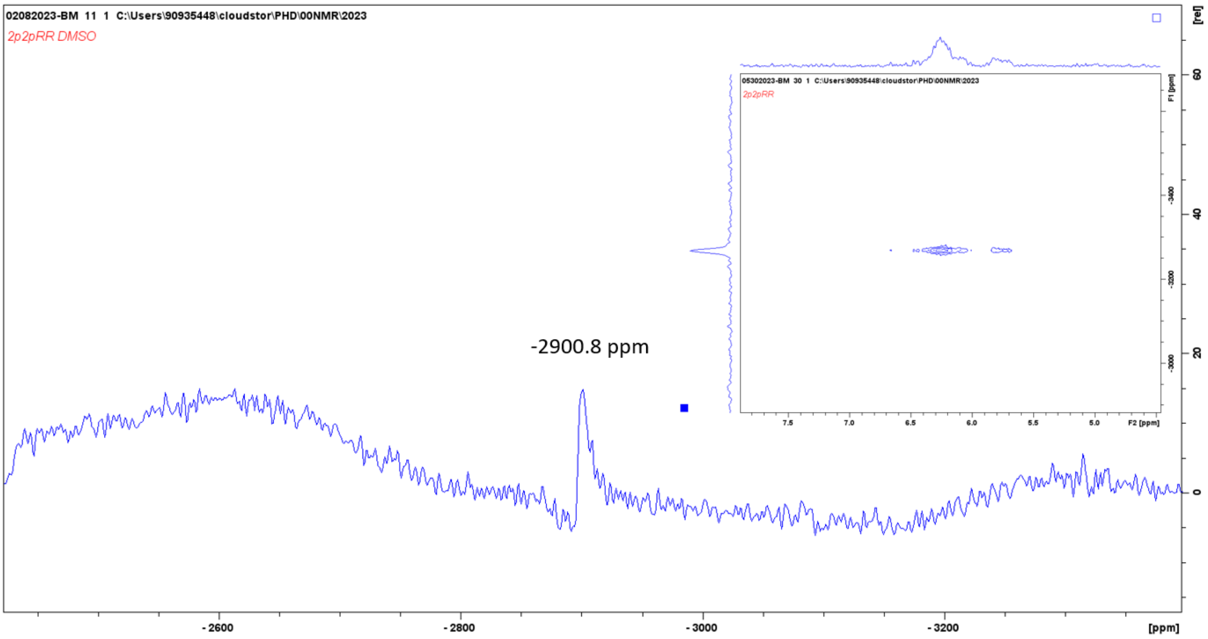

Figure S14: ^{195}Pt peak spectra of 4b [Pt(PyPy)(*RR*DACH)]$^{2+}$ in DMSO, spectral width of 214436 Hz and 256 data points for the ^{195}Pt nucleus (F1 dimension) and a spectral width of 4808 Hz with 2048 data points for the ^{1}H nucleus (F2 dimension).

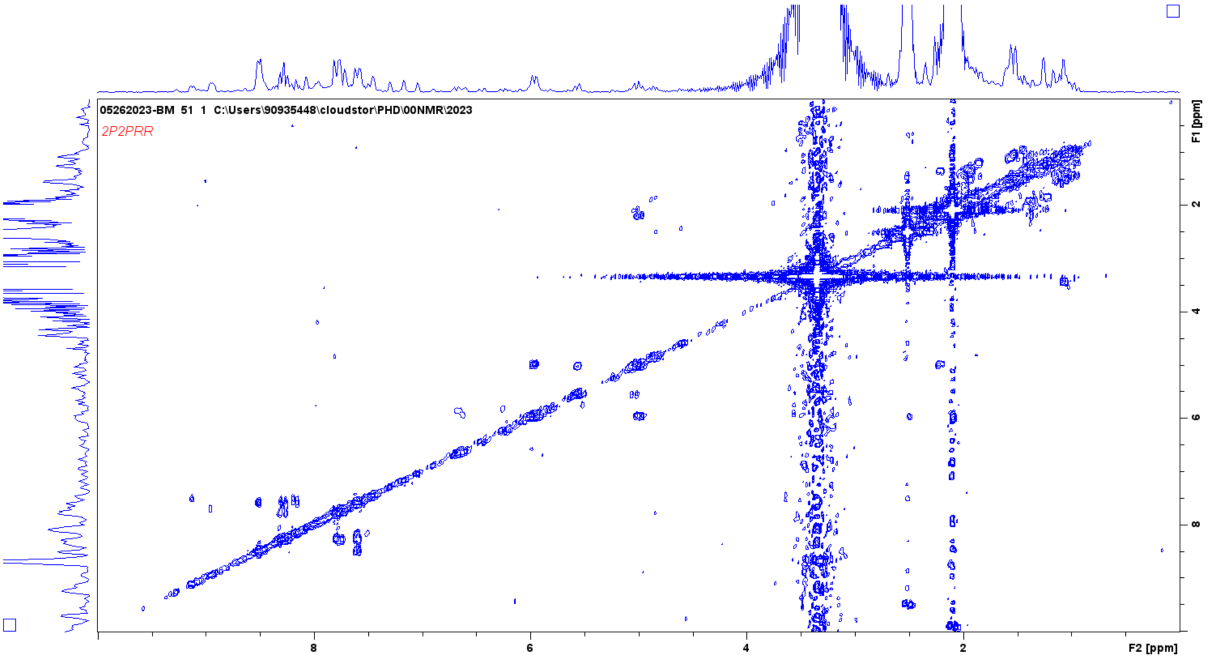

Figure S15: ^{1}H/ ^{1}H COSY spectra of 4b [Pt(PyPy)(*RR*DACH)]$^{2+}$ in DMSO, F1/F2 ^{1}H dimension spectral width of 8250 Hz and 65536 data points.

[Pt(ImPy)(*SS*DACH)]$^{2+}$

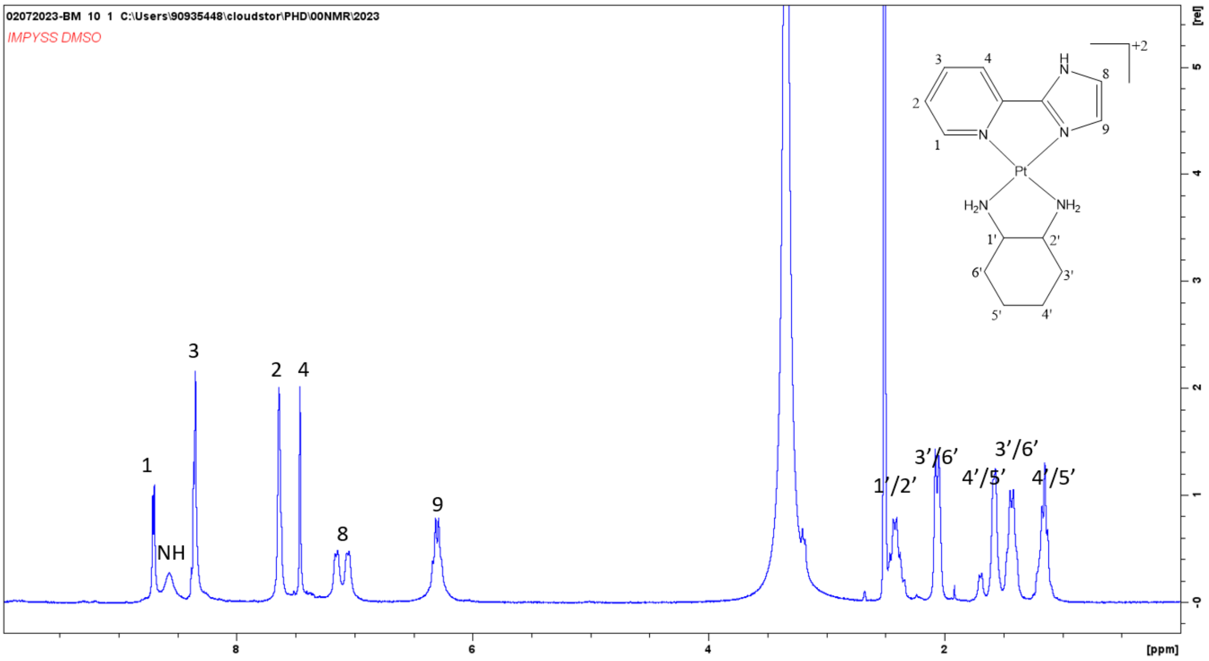

Figure S16: ^{1}H NMR spectra of 5a [Pt(ImPy)(*SS*DACH)]$^{2+}$ in DMSO, spectral width of 8250 Hz and 65536 data points.

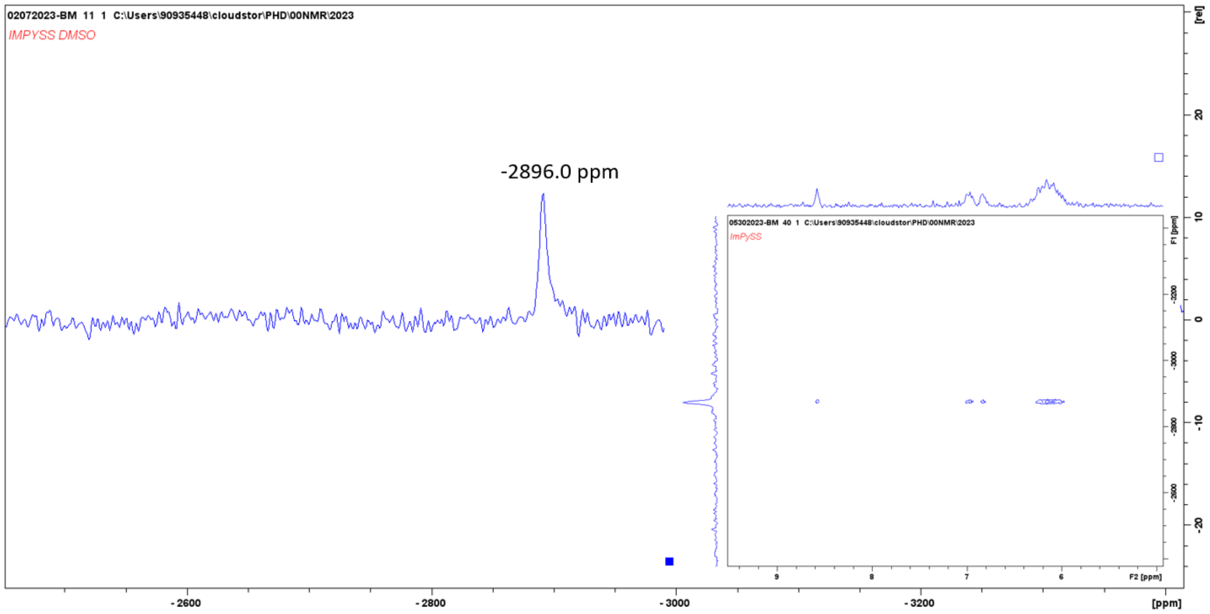

Figure S17: ^{195}Pt peak spectra of 5a $[Pt(ImPy)(\textit{SS}DACH)]^{2+}$ in DMSO, spectral width of 8250 Hz and 65536 data points.

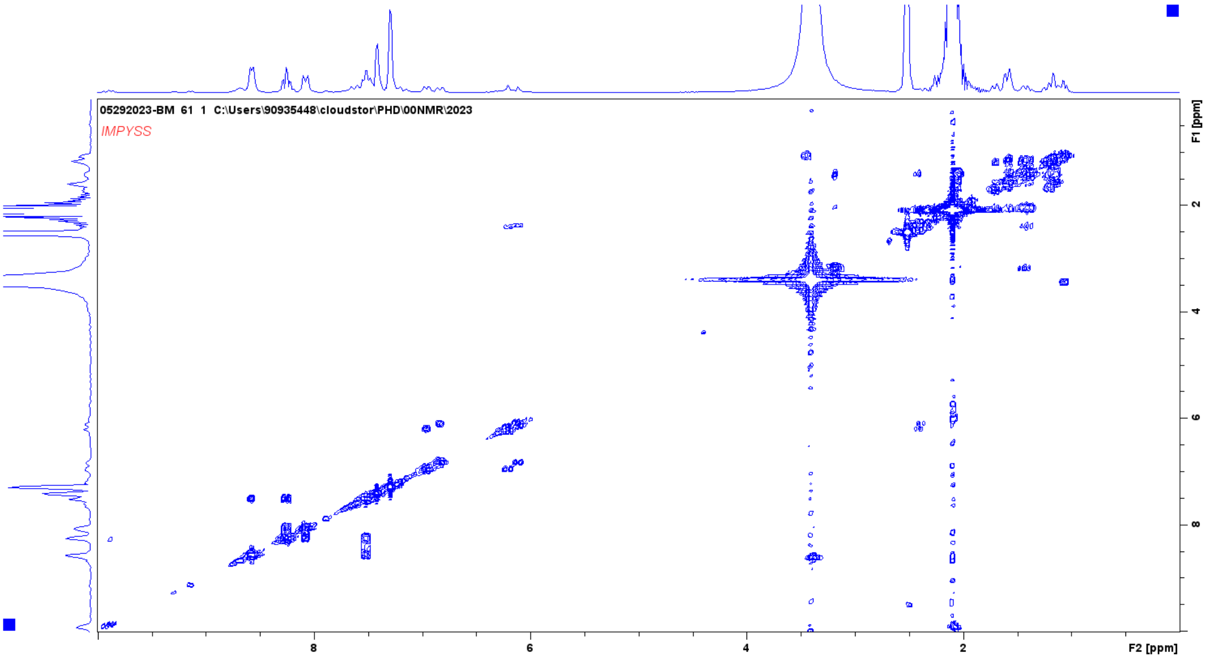

Figure S18: ^{1}H/ ^{1}H COSY spectra of 5a $[Pt(ImPy)(\textit{SS}DACH)]^{2+}$ in DMSO, F1/F2 ^{1}H dimension spectral width of 8250 Hz and 65536 data points.

[Pt(ImPy)(*RR*DACH)]$^{2+}$

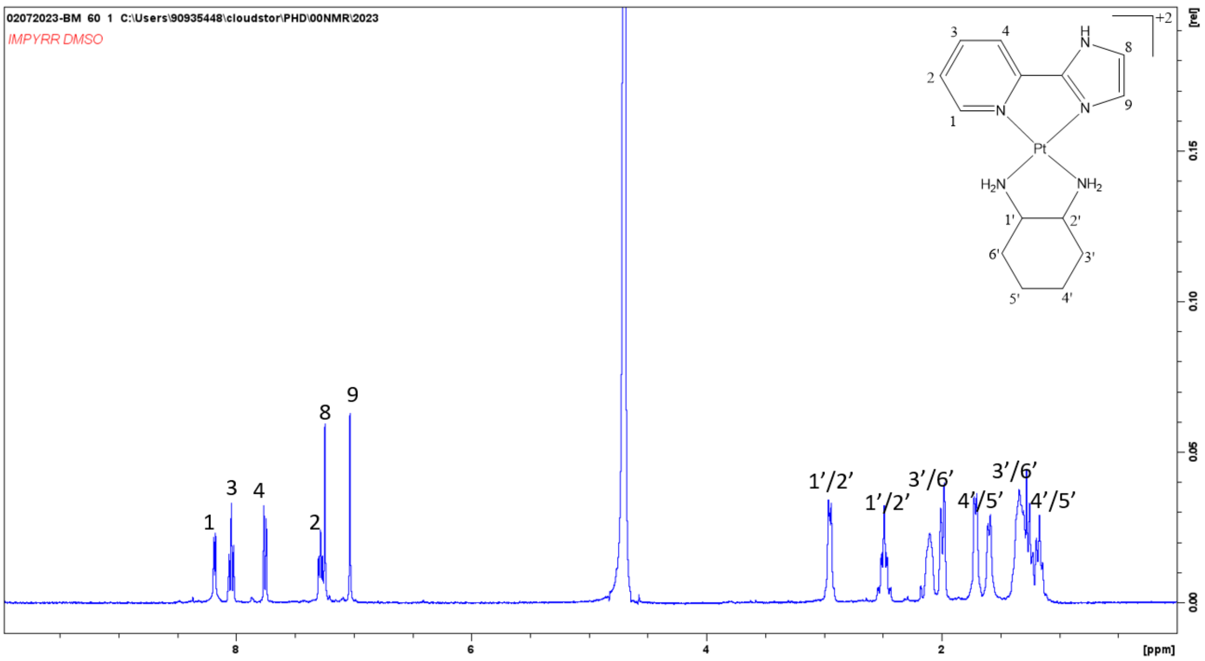

Figure S19: ^{1}H NMR spectra of 5b [Pt(ImPy)(*RR*DACH)]$^{2+}$ in DMSO, spectral width of 8250 Hz and 65536 data points.

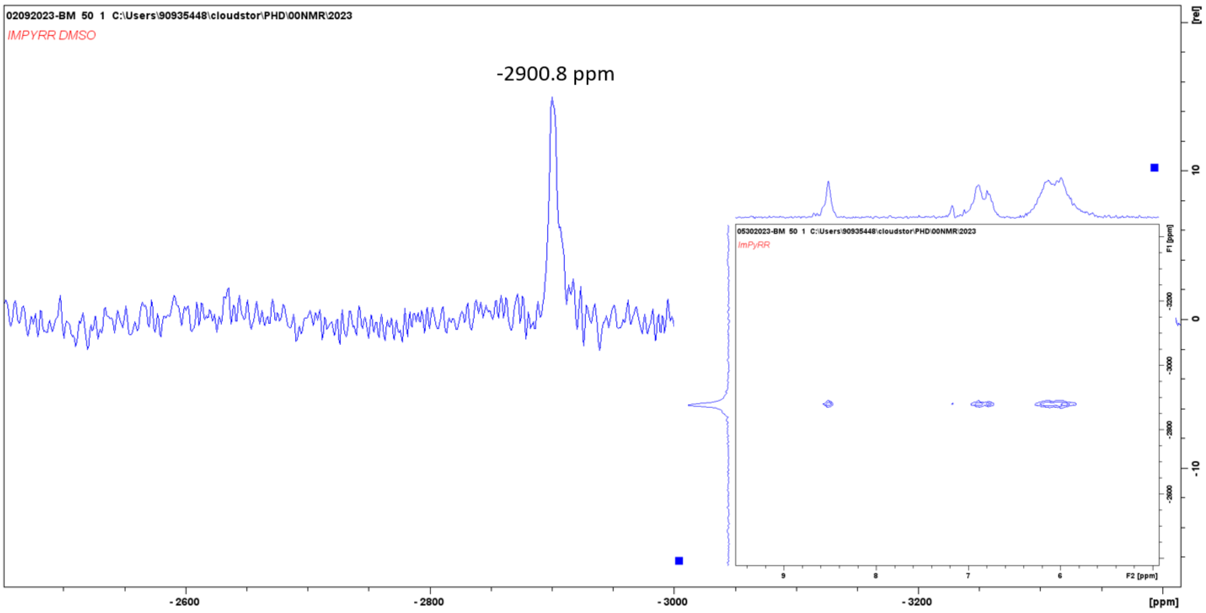

Figure S20: ^{195}Pt peak spectra of 5b [Pt(ImPy)(*RR*DACH)]$^{2+}$ in DMSO, spectral width of 214436 Hz and 256 data points for the ^{195}Pt nucleus (F1 dimension) and a spectral width of 4808 Hz with 2048 data points for the ^{1}H nucleus (F2 dimension).

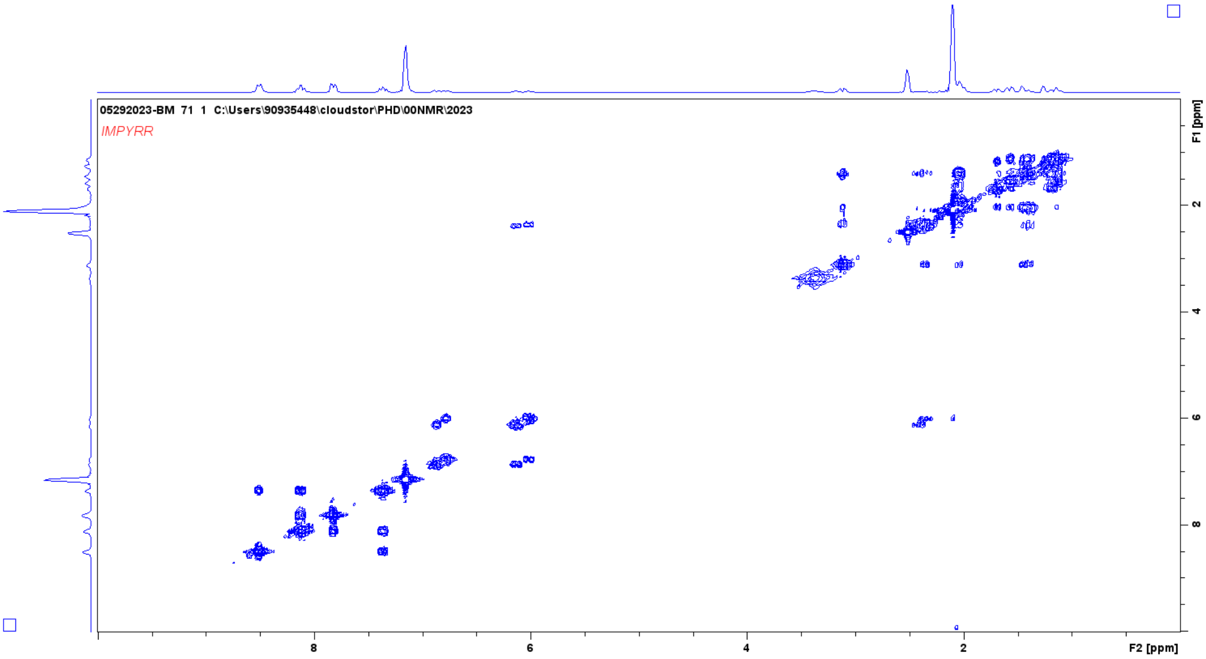

Figure S21: ^{1}H/ ^{1}H COSY spectra of 5b [Pt(ImPy)(*RR*DACH)]$^{2+}$ in DMSO, F1/F2 ^{1}H dimension spectral width of 8250 Hz and 65536 data points.

[Pt(BImPy)(*SS*DACH)]$^{2+}$

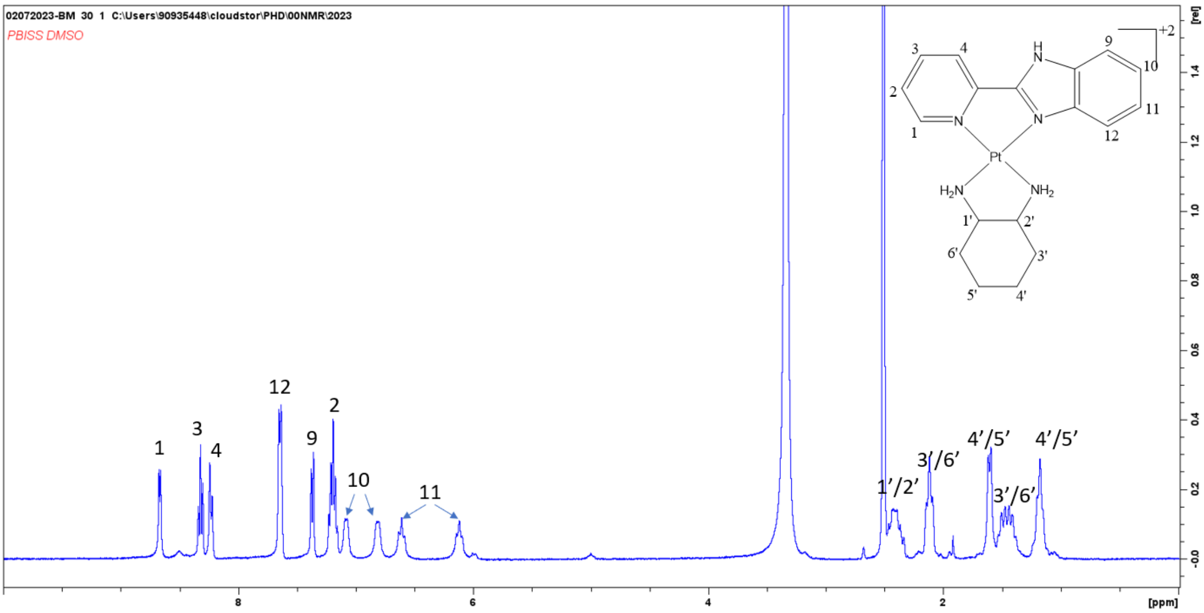

Figure S22: ^{1}H NMR spectra of 5b[Pt(BImPy)(*SS*DACH)]$^{2+}$ in DMSO, spectral width of 8250 Hz and 65536 data points.

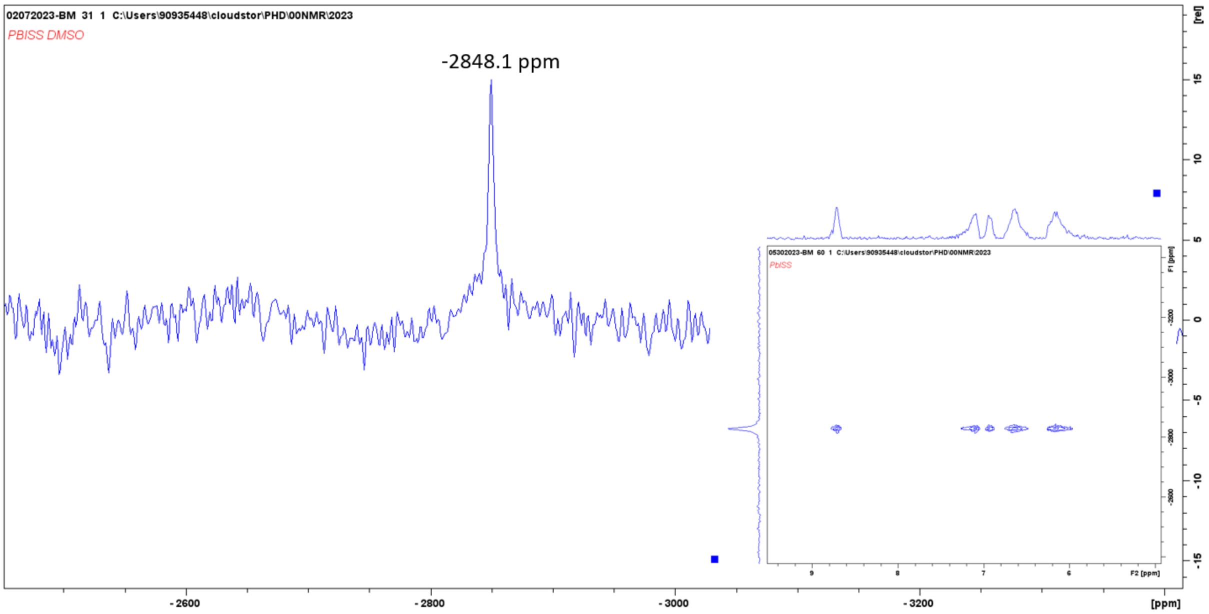

Figure S23: ^{195}Pt peak spectra of 6a [Pt(BImPy)(*SS*DACH)]$^{2+}$ in DMSO, spectral width of 214436 Hz and 256 data points for the ^{195}Pt nucleus (F1 dimension) and a spectral width of 4808 Hz with 2048 data points for the ^{1}H nucleus (F2 dimension).

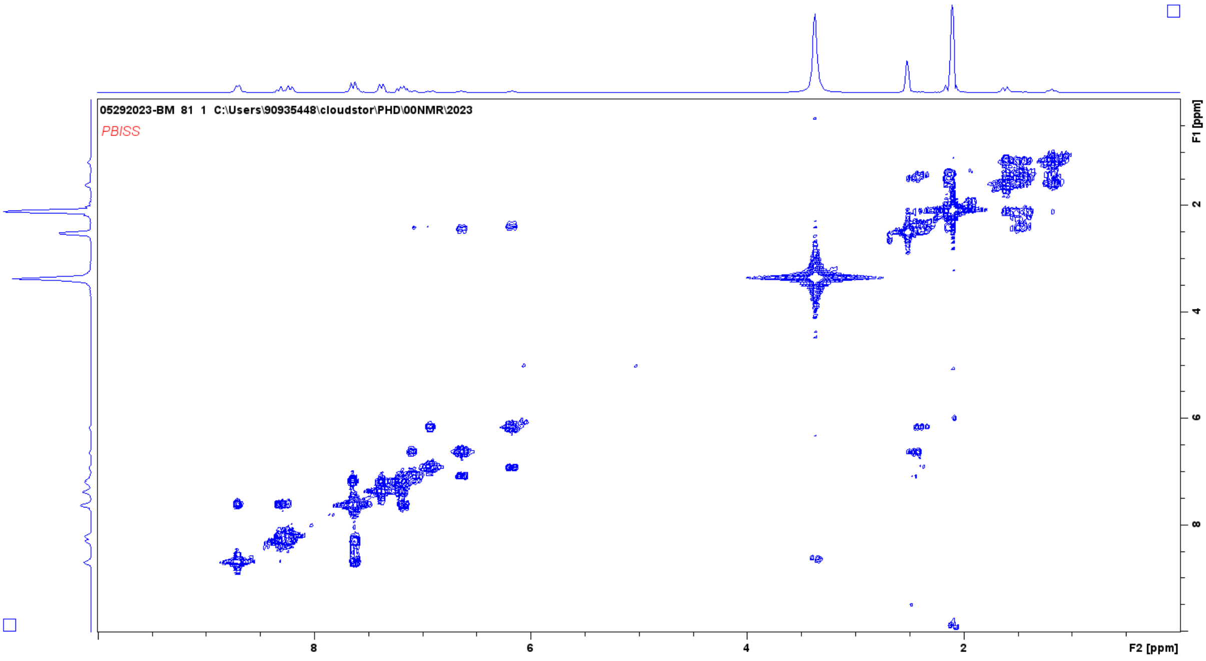

Figure S24: ^{1}H/ ^{1}H COSY spectra of 6a [Pt(BImPy)(*SS*DACH)]$^{2+}$ in DMSO, F1/F2 ^{1}H dimension spectral width of 8250 Hz and 65536 data points.

[Pt(BImPy)(*RR*DACH)]$^{2+}$

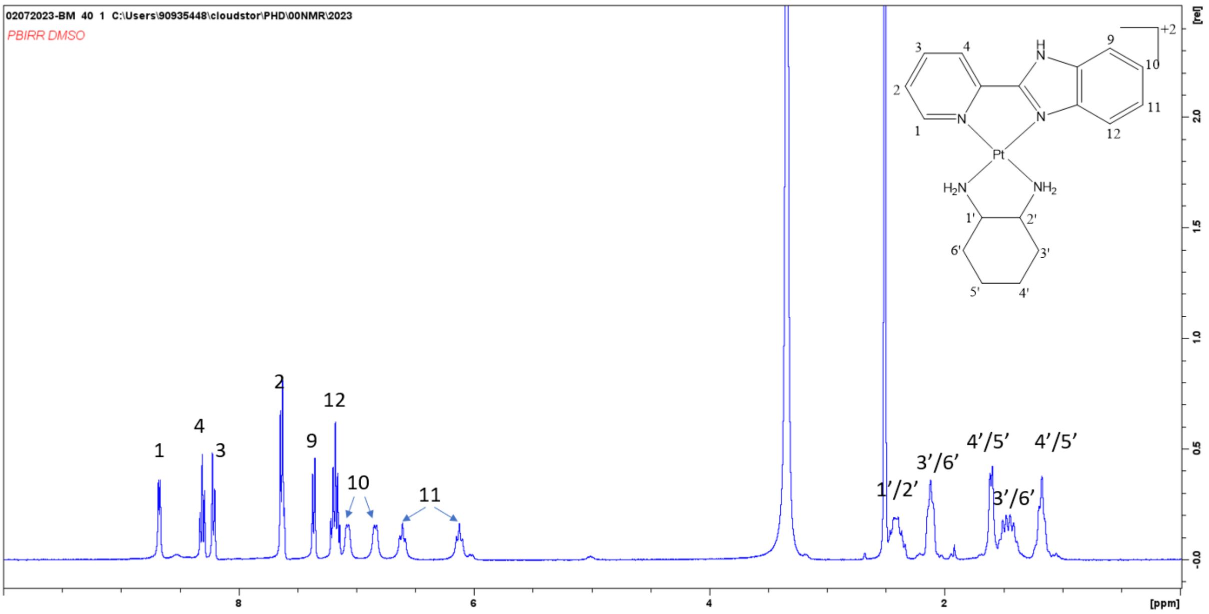

Figure S25: ^{1}H NMR spectra of 6b [Pt(BImPy)(*SS*DACH)]$^{2+}$ in DMSO, spectral width of 8250 Hz and 65536 data points.

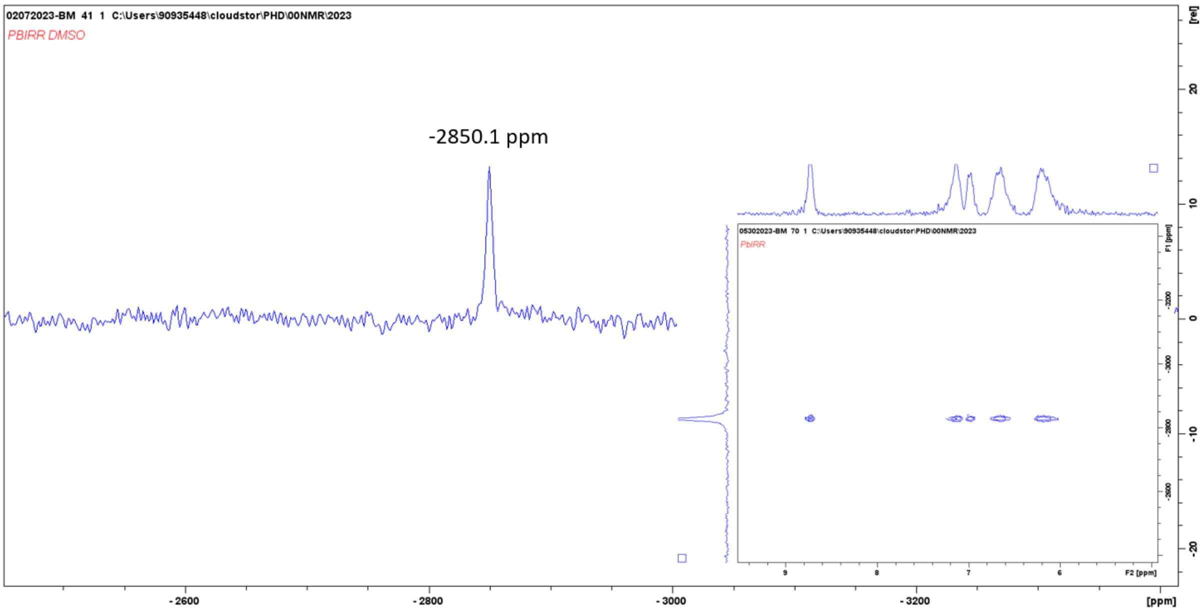

Figure S26: ^{195}Pt peak spectra of 6b [Pt(BImPy)(*SS*DACH)]$^{2+}$ in DMSO, spectral width of 214436 Hz and 256 data points for the ^{195}Pt nucleus (F1 dimension) and a spectral width of 4808 Hz with 2048 data points for the ^{1}H nucleus (F2 dimension).

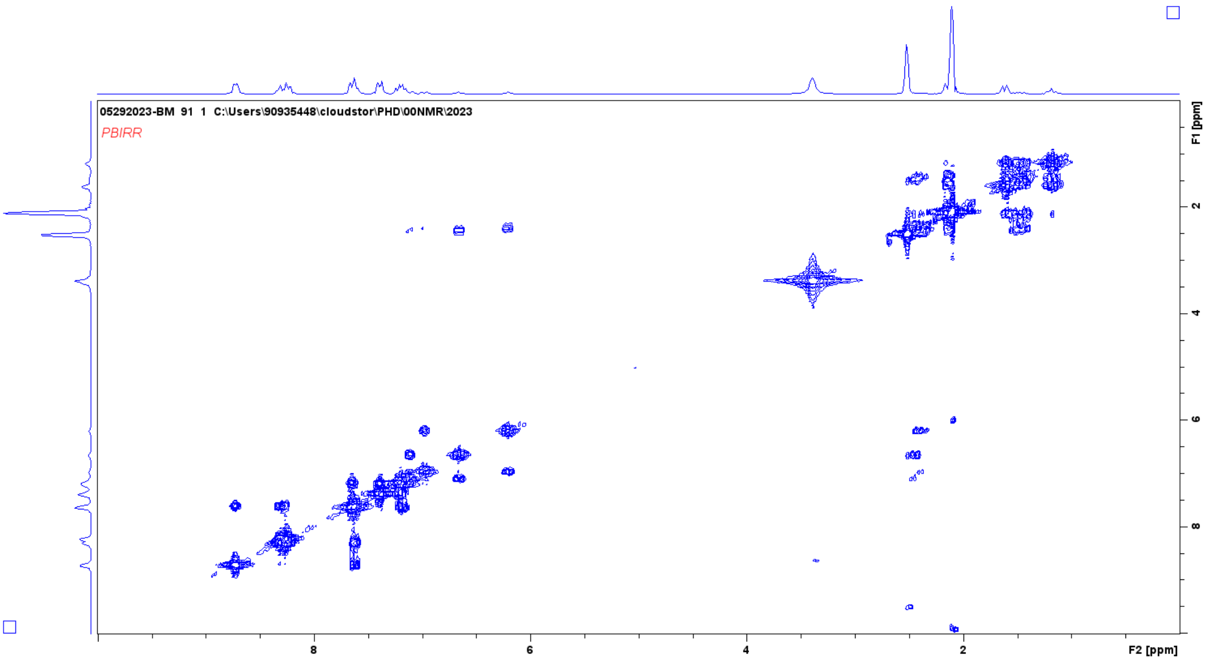

Figure S27: ^{1}H/ ^{1}H COSY spectra of 6b [Pt(BImPy)(*RR*DACH)]$^{2+}$ in DMSO, F1/F2 ^{1}H dimension spectral width of 8250 Hz and 65536 data points.

Molar Absorption Coefficients

[Pt(PyPy)(*SS*DACH)]$^{2+}$

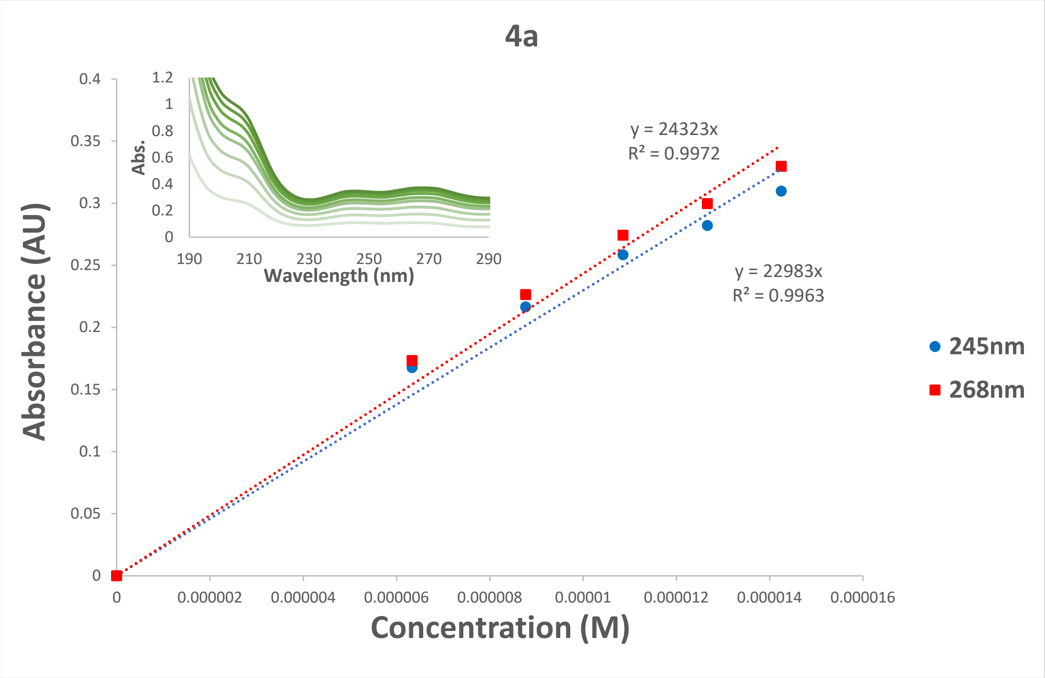

Figure S28: Titration of a stock solution of 4a [Pt(PyPy)(*SS*DACH)]$^{2+}$ into a known concentration in H_2O and the resulting extinction coefficient calculated based on the two main peaks at 241 nm (red) and 287 nm (blue).

[Pt(PyPy)(*RR*DACH)]$^{2+}$

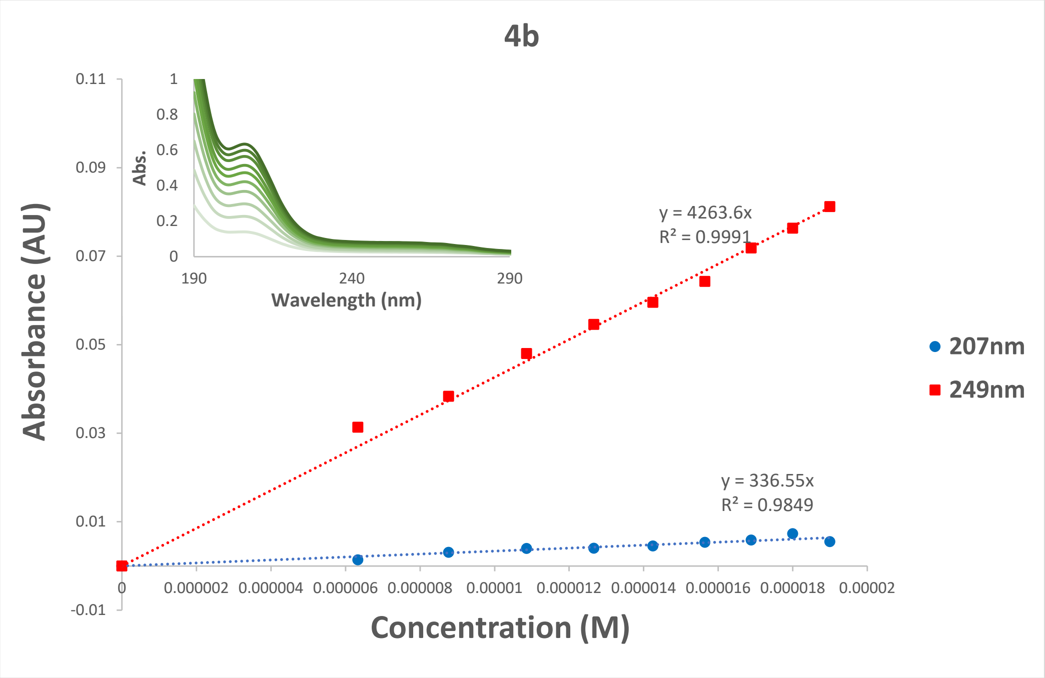

Figure S29: Titration of a stock solution of 4b [Pt(PyPy)(*RR*DACH)]$^{2+}$ into a known concentration in H_2O and the resulting extinction coefficient calculated based on the two main peaks at 241 nm (red) and 287 nm (blue).

[Pt(ImPy)(*SS*DACH)]$^{2+}$

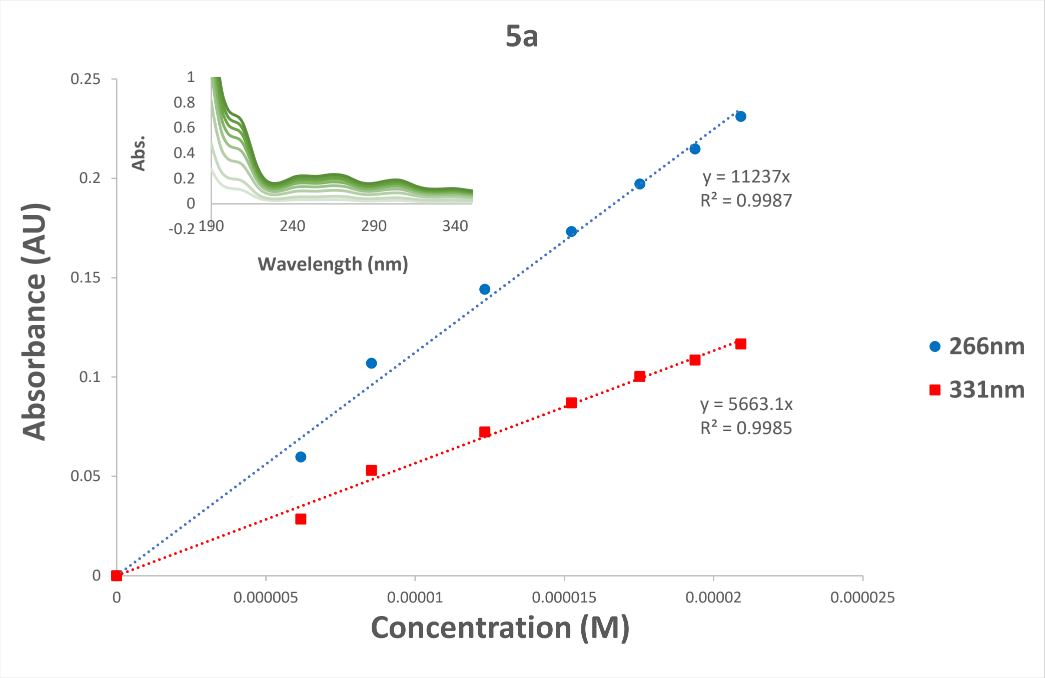

Figure S30: Titration of a stock solution of 5a [Pt(ImPy)(*SS*DACH)]$^{2+}$ into a known concentration in H_2O and the resulting extinction coefficient calculated based on the two main peaks at 241 nm (red) and 287 nm (blue).

[Pt(ImPy)(*RR*DACH)]$^{2+}$

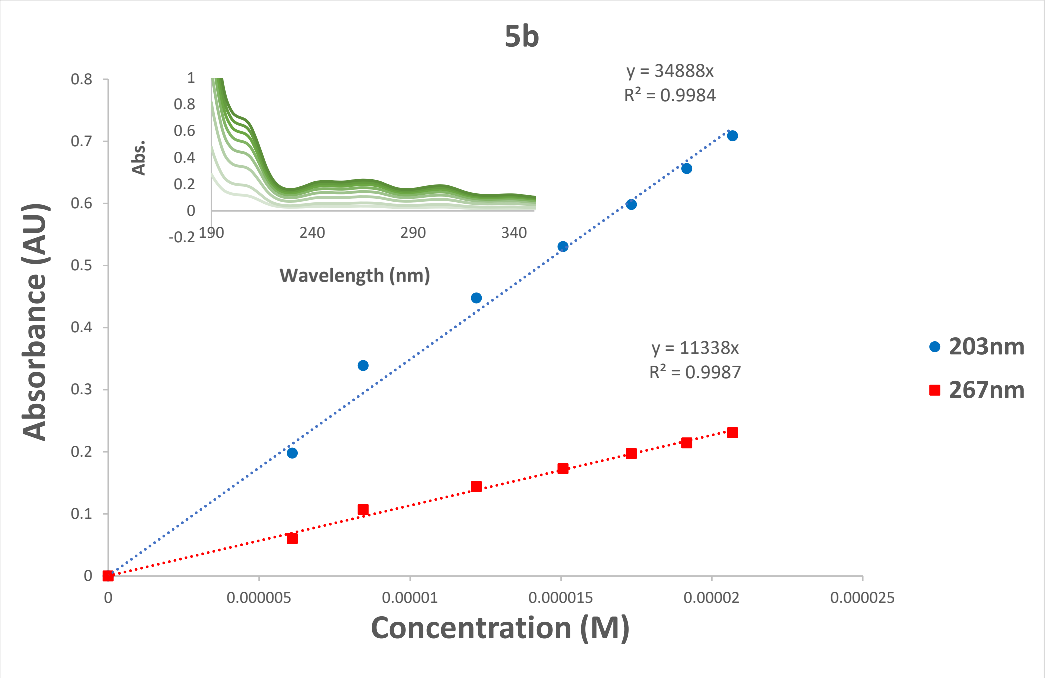

Figure S31: Titration of a stock solution of 5b [Pt(ImPy)(*RR*DACH)]$^{2+}$ into a known concentration in H_2O and the resulting extinction coefficient calculated based on the two main peaks at 241 nm (red) and 287 nm (blue).

[Pt(BImPy)(*SS*DACH)]$^{2+}$

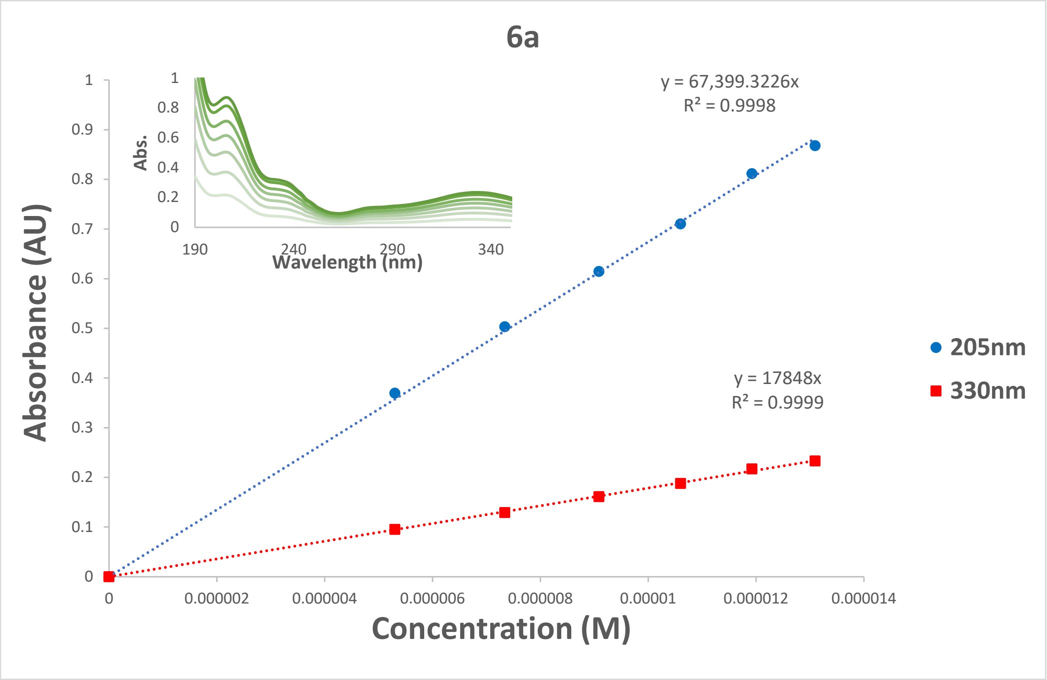

Figure S32: Titration of a stock solution of 6a [Pt(BImPy)(*SS*DACH)]$^{2+}$ into a known concentration in H_2O and the resulting extinction coefficient calculated based on the two main peaks at 330 nm (red) and 205 nm (blue).

[Pt(BImPy)(*RR*DACH)]$^{2+}$

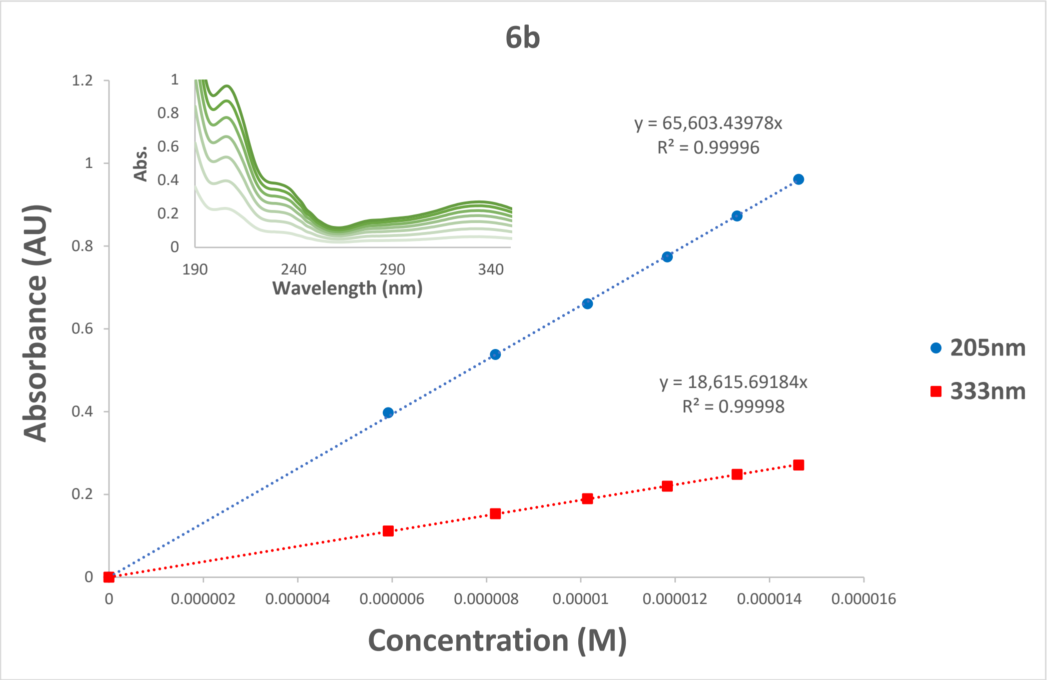

Figure S33: Titration of a stock solution of 6b[Pt(BImPy)(*RR*DACH)]$^{2+}$ into a known concentration in H_2O and the resulting extinction coefficient calculated based on the two main peaks at 241 nm (red) and 287 nm (blue).

HPLC

All HPLC's show purity over 95%

[Pt(PyPy)(*SS*DACH)]$^{2+}$

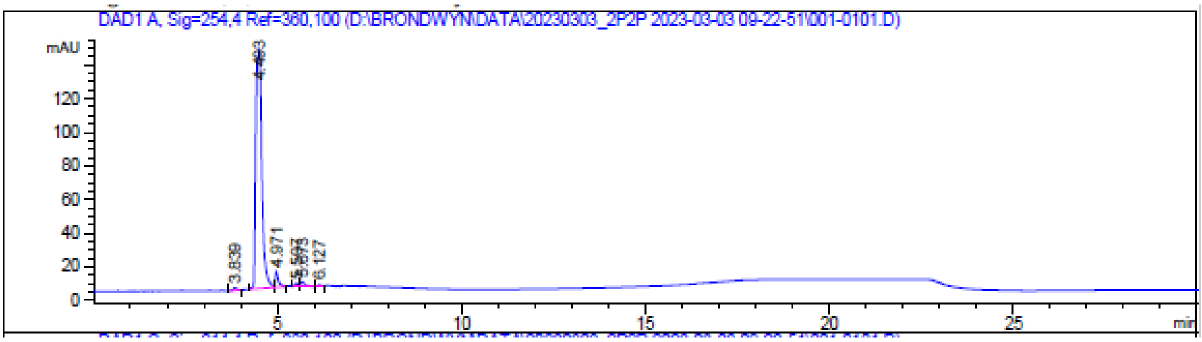

Figure S34. HPLC spectrum of 4a ([Pt(2P2P)(*SS*DACH)]$^{2+}$) measured on a Agilent Technologies 1260 Infinity machine equipped with a Phenomenex Onyx™ Monolithic C18 reverse phase column (100 × 4.6 mm, 130 Å). Sample solutions were made up in H_2O and injected at a 0-100 gradient A to B over 15 minutes with a 15 minute flush in-between samples.

[Pt(ImPy)(*SS*DACH)]$^{2+}$

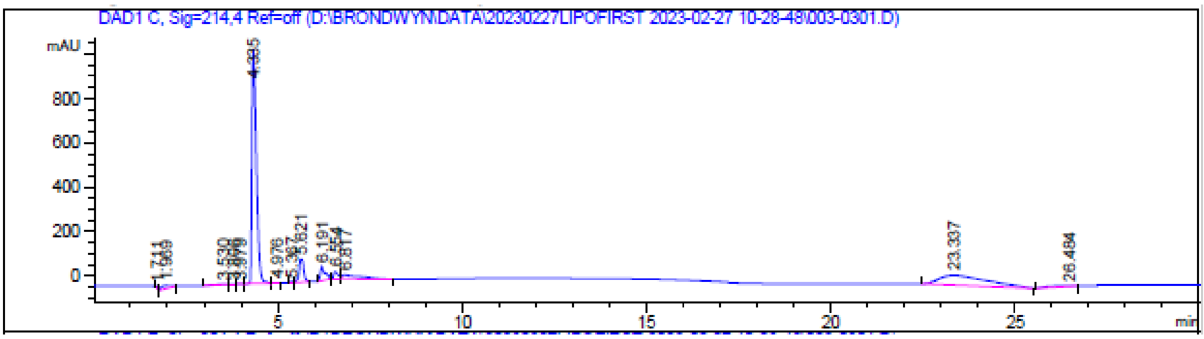

Figure S35. HPLC spectrum of 4b ([Pt(ImPy)(*SS*DACH)]$^{2+}$) measured on a Agilent Technologies 1260 Infinity machine equipped with a Phenomenex Onyx™ Monolithic C18 reverse phase column (100 × 4.6 mm, 130 Å). Sample solutions were made up in H_2O and injected at a 0-100 gradient A to B over 15 minutes with a 15 minute flush in-between samples.

[Pt(BImPy)(*SS*DACH)]$^{2+}$

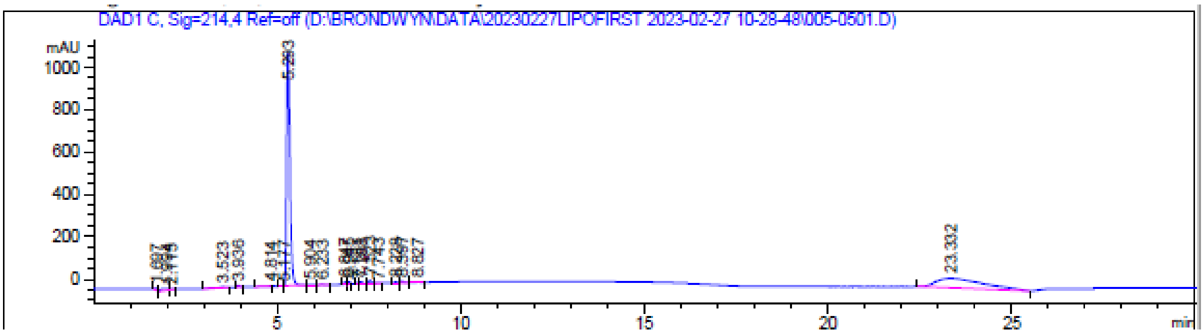

Figure S36. HPLC spectrum of 5a ([Pt(BImPy)(*SS*DACH)]$^{2+}$) measured on a Agilent Technologies 1260 Infinity machine equipped with a Phenomenex Onyx™ Monolithic C18 reverse phase column (100 × 4.6 mm, 130 Å). Sample solutions were made up in H_2O and injected at a 0-100 gradient A to B over 15 minutes with a 15 minute flush in-between samples.

[Pt(PyPy)(*RR*DACH)]$^{2+}$

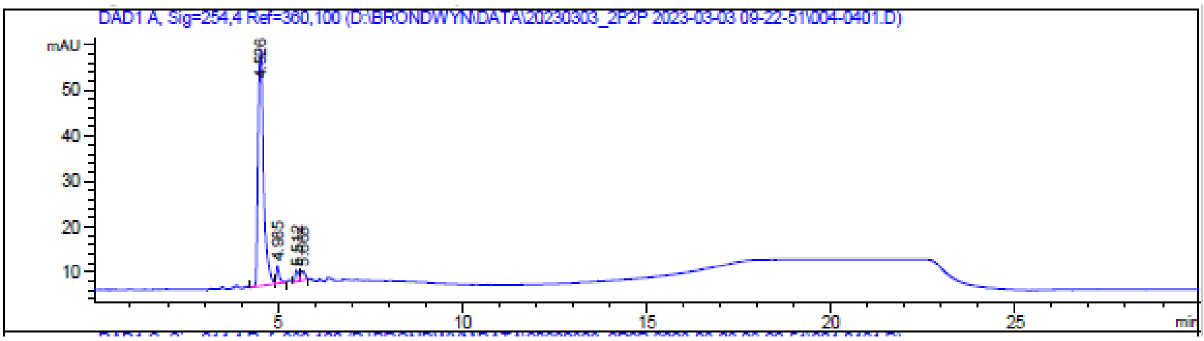

Figure S37. HPLC spectrum of 5b ([Pt(2P2P)(*RR*DACH)]$^{2+}$) measured on a Agilent Technologies 1260 Infinity machine equipped with a Phenomenex Onyx™ Monolithic C18 reverse phase column (100 × 4.6 mm, 130 Å). Sample solutions were made up in H_2O and injected at a 0-100 gradient A to B over 15 minutes with a 15 minute flush in-between samples.

[Pt(ImPy)(*RR*DACH)]$^{2+}$

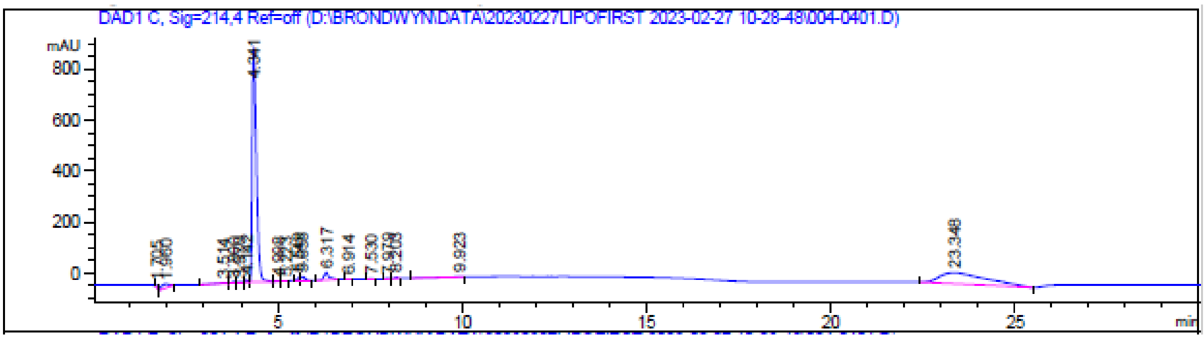

Figure S38. HPLC spectrum of 6a ([Pt(ImPy)(*RR*DACH)]$^{2+}$) measured on a Agilent Technologies 1260 Infinity machine equipped with a Phenomenex Onyx™ Monolithic C18 reverse phase column (100 × 4.6 mm, 130 Å). Sample solutions were made up in H_2O and injected at a 0-100 gradient A to B over 15 minutes with a 15 minute flush in-between samples.

[Pt(BImPy)(*RR*DACH)]$^{2+}$

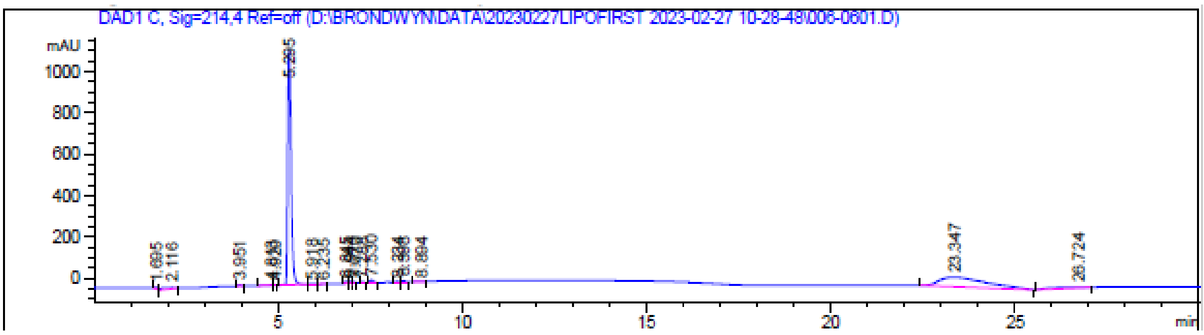

Figure S39. HPLC spectrum of 6b ([Pt(BImPy)(*RR*DACH)]$^{2+}$) measured on a Agilent Technologies 1260 Infinity machine equipped with a Phenomenex Onyx™ Monolithic C18 reverse phase column (100 × 4.6 mm, 130 Å). Sample solutions were made up in H_2O and injected at a 0-100 gradient A to B over 15 minutes with a 15 minute flush in-between samples.

CD

[Pt(PyPy)(*SS*DACH)]$^{2+}$

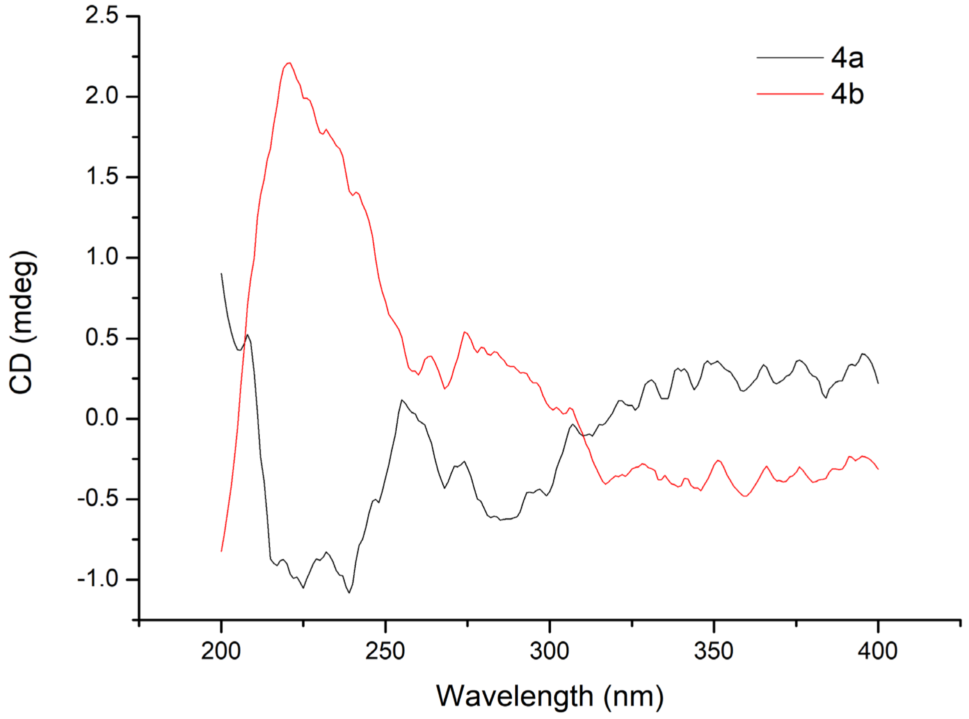

Figure S40: CD spectra of **4a** ([Pt(2P2P)(*SS*DACH)]$^{2+}$) (black) and **4b** ([Pt(2P2P)(*RR*DACH)]$^{2+}$) (red) at room temperature in the 200–400 nm range, using a 10 mm quartz cell, corrected for solvent baseline, measured in H_2O

[Pt(ImPy)(*SS*DACH)]$^{2+}$

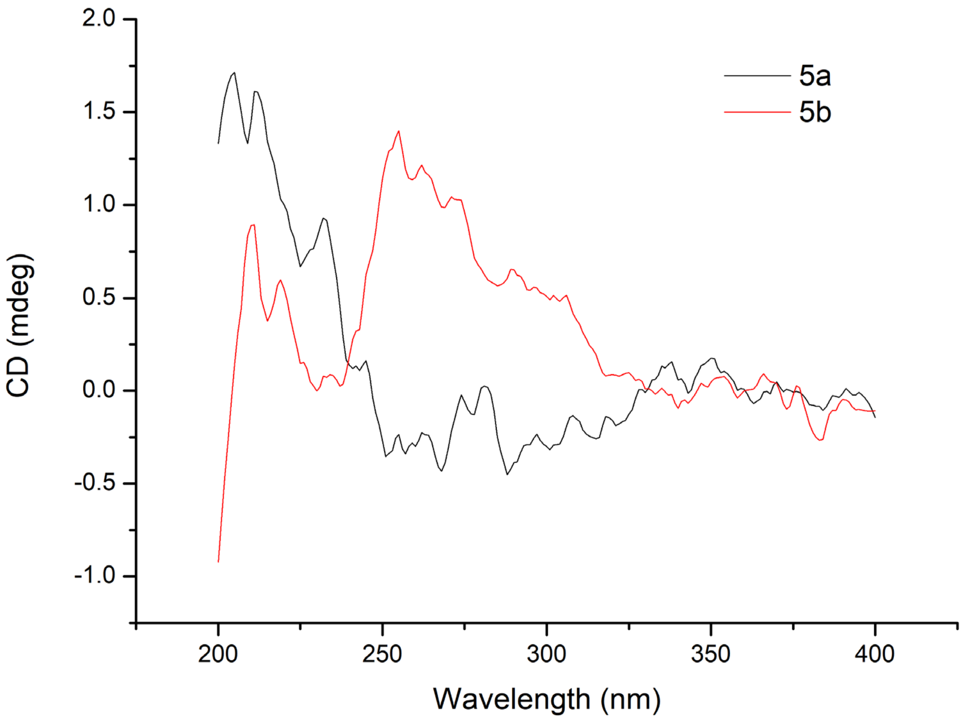

Figure S41: CD spectra of **5a** ([Pt(ImPy)(*SS*DACH)]$^{2+}$) (black) and **5b** ([Pt(ImPy)(*RR*DACH)]$^{2+}$) (red) at room temperature in the 200–400 nm range, using a 10 mm quartz cell, corrected for solvent baseline, measured in H_2O

[Pt(BImPy)(*SS*DACH)]$^{2+}$

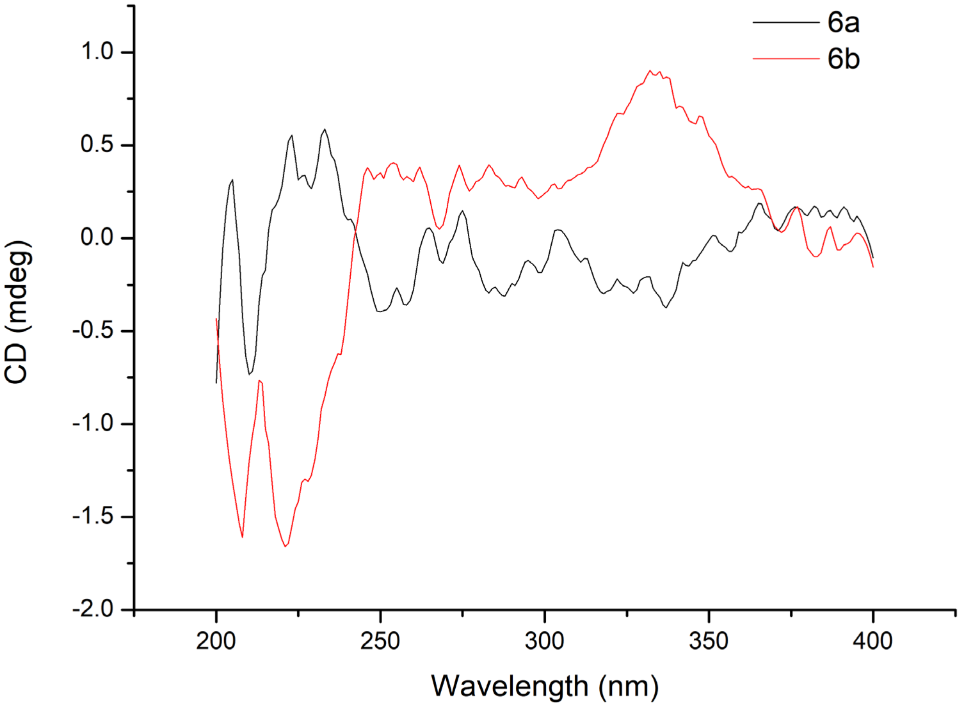

Figure S42: CD spectra of **6a** ([Pt(BImPy)(*SS*DACH)]$^{2+}$) (black) and **6b** ([Pt(BImPy)(*RR*DACH)]$^{2+}$) (red) at room temperature in the 200–400 nm range, using a 10 mm quartz cell, corrected for solvent baseline, measured in H_2O

ESI MS

[Pt(PyPy)(*SS*DACH)]$^{2+}$

Elemental Composition Report **Page 1**

Multiple Mass Analysis: 2 mass(es) processed
Tolerance = 20.0 PPM / DBE: min = -1.5, max = 50.0
Element prediction: Off
Number of isotope peaks used for i-FIT = 9

Monoisotopic Mass, Even Electron Ions
140 formula(e) evaluated with 2 results within limits (all results (up to 1000) for each mass)
Elements Used:
C: 0-27 H: 0-35 N: 0-5 Na: 0-1 Br: 0-1 Pt: 1-1

230403 1 32 (0.637) AM2 (Ar,20000.0,556.28,0.00,LS 3); Cm (27:41)
1: TOF MS ES+

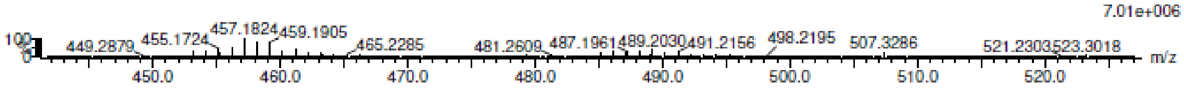

Minimum:	80.00				-1.5				
Maximum:	100.00		20.0	20.0	50.0				
Mass	RA	Calc. Mass	mDa	PPM	DBE	i-FIT	Norm	Conf(%)	Formula
457.1824	100.00	457.1907	-8.3	-18.2	2.5	1888.3	n/a	n/a	C14 H29 N3 Na Pt
459.1905	80.25	459.1836	6.9	15.0	5.5	1959.3	n/a	n/a	C14 H26 N5 Pt

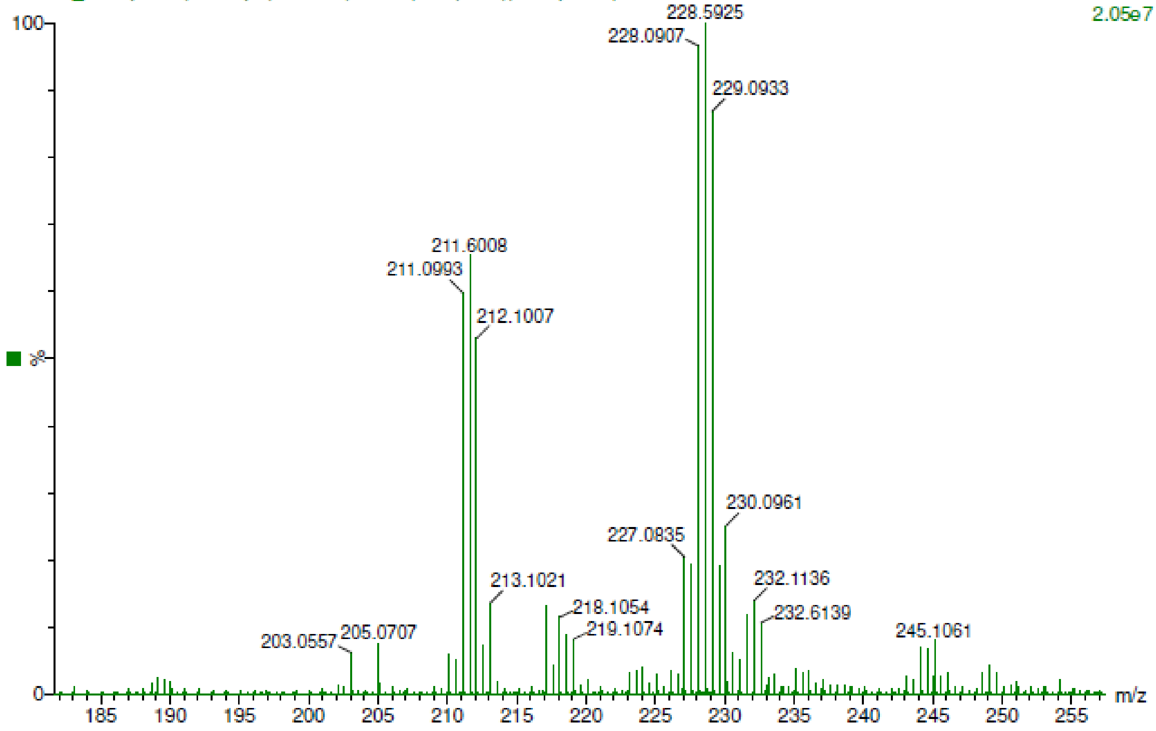

Figure S43. ESIMS spectrum of [Pt(PyPy)(*SS*DACH)]$^{+2}$ measured on a Waters TQ-MS triple quadrupole mass spectrometer. Sample solutions were made up to 0.3 mM in H_2O: MeOH (90:10) and run at 0.1 mL/min.

[Pt(PyPy)(*RR*DACH)]$^{2+}$

Elemental Composition Report Page 1

Multiple Mass Analysis: 2 mass(es) processed
Tolerance = 20.0 PPM / DBE: min = -1.5, max = 50.0
Element prediction: Off
Number of isotope peaks used for i-FIT = 9

Monoisotopic Mass, Even Electron Ions
140 formula(e) evaluated with 2 results within limits (all results (up to 1000) for each mass)
Elements Used:
C: 0-27 H: 0-35 N: 0-5 Na: 0-1 Br: 0-1 Pt: 1-1

230403 1 32 (0.637) AM2 (Ar,20000.0,556.28,0.00,LS 3); Cm (27:41)
1: TOF MS ES+

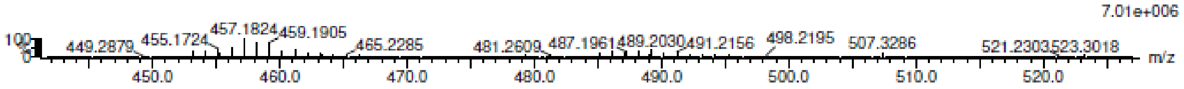

Minimum:	80.00				-1.5				
Maximum:	100.00		20.0	20.0	50.0				
Mass	RA	Calc. Mass	mDa	PPM	DBE	i-FIT	Norm	Conf(%)	Formula
457.1824	100.00	457.1907	-8.3	-18.2	2.5	1888.3	n/a	n/a	C14 H29 N3 Na Pt
459.1905	80.25	459.1836	6.9	15.0	5.5	1959.3	n/a	n/a	C14 H26 N5 Pt

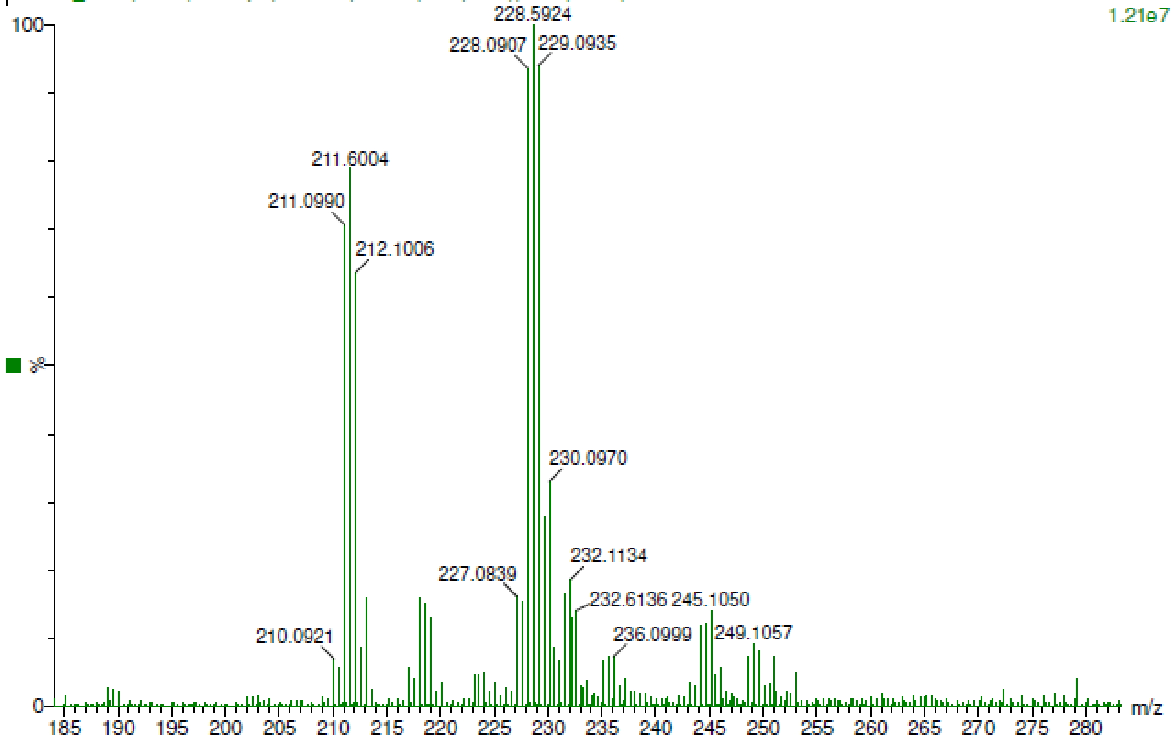

Figure S44. ESIMS spectrum of [Pt(PyPy)(*RR*DACH)]$^{+2}$ measured on a Waters TQ-MS triple quadrupole mass spectrometer. Sample solutions were made up to 0.3 mM in H_2O: MeOH (90:10) and run at 0.1 mL/min.

[Pt(ImPy)(*SS*DACH)]$^{2+}$

Elemental Composition Report

Page 1

Single Mass Analysis

Tolerance = 20.0 PPM / DBE: min = -1.5, max = 50.0
Element prediction: Off
Number of isotope peaks used for i-FIT = 9

Monoisotopic Mass, Even Electron Ions
72 formula(e) evaluated with 2 results within limits (all results (up to 1000) for each mass)
Elements Used:
C: 0-20 H: 0-30 N: 0-6 Na: 0-1 Br: 0-1 Pt: 1-1

230403 3 46 (0.913) AM2 (Ar,20000.0,556.28,0.00,LS 3)
1: TOF MS ES+

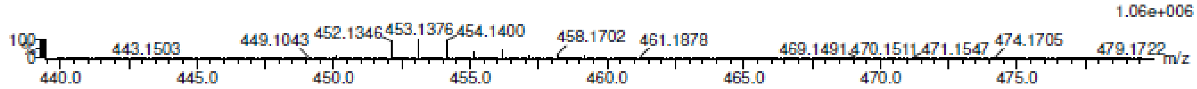

Mass	Calc. Mass	mDa	PPM	DBE	i-FIT	Norm	Conf(%)	Formula
Minimum:				-1.5				
Maximum:		20.0	20.0	50.0				
453.1376	453.1366	1.0	2.2	8.5	1622.0	0.553	57.54	C14 H20 N5 Pt
	453.1342	3.4	7.5	5.5	1622.3	0.857	42.46	C12 H21 N5 Na Pt

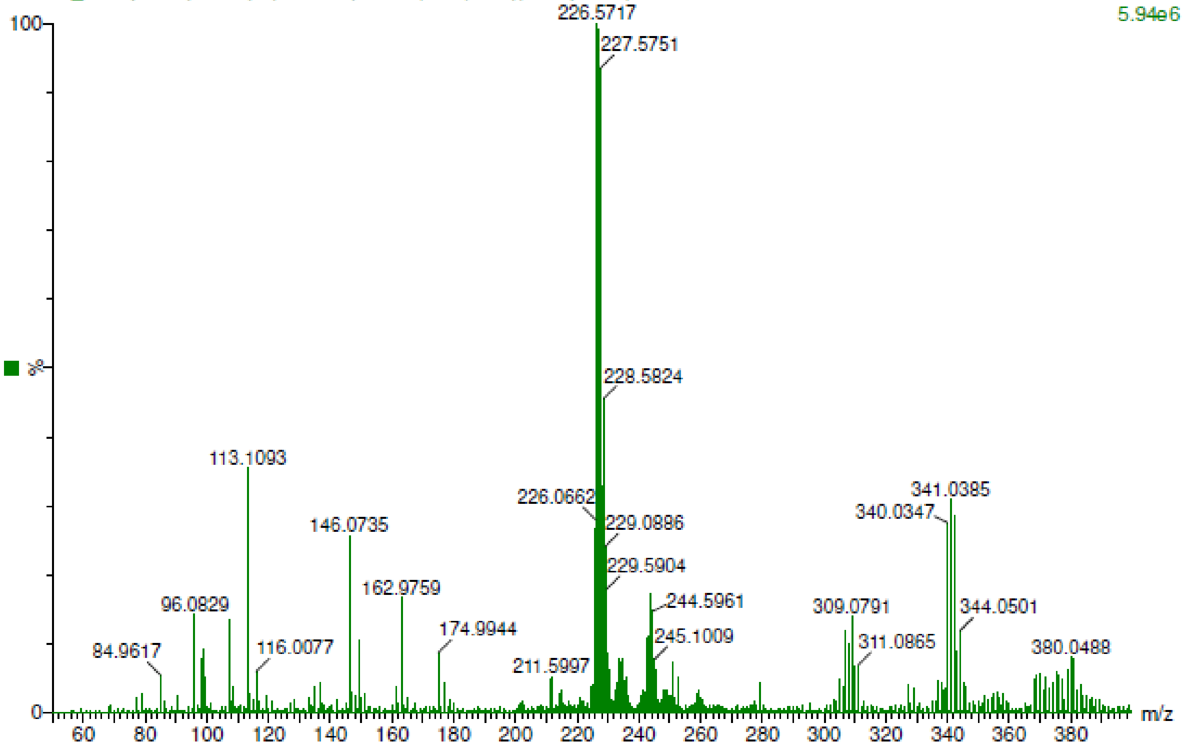

Figure S45. ESIMS spectrum of [Pt(ImPy)(*SS*DACH)]$^{+2}$ measured on a Waters TQ-MS triple quadrupole mass spectrometer. Sample solutions were made up to 0.3 mM in H_2O: MeOH (90:10) and run at 0.1 mL/min.

[Pt(ImPy)(*RR*DACH)]$^{2+}$

Elemental Composition Report

Page 1

Single Mass Analysis

Tolerance = 20.0 PPM / DBE: min = -1.5, max = 50.0
Element prediction: Off

Monoisotopic Mass, Even Electron Ions
72 formula(e) evaluated with 2 results within limits (all results (up to 1000) for each mass)
Elements Used:
C: 0-20 H: 0-30 N: 0-6 Na: 0-1 Br: 0-1 Pt: 1-1

230403_4 41 (0.829) Cm (41:46)
1: TOF MS ES+

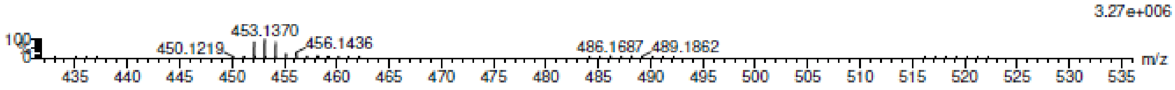

	Mass	Calc. Mass	mDa	PPM	DBE	Formula
Minimum:					-1.5	
Maximum:			20.0	20.0	50.0	
	453.1370	453.1366	0.4	0.9	8.5	C14 H20 N5 Pt
		453.1342	2.8	6.2	5.5	C12 H21 N5 Na Pt

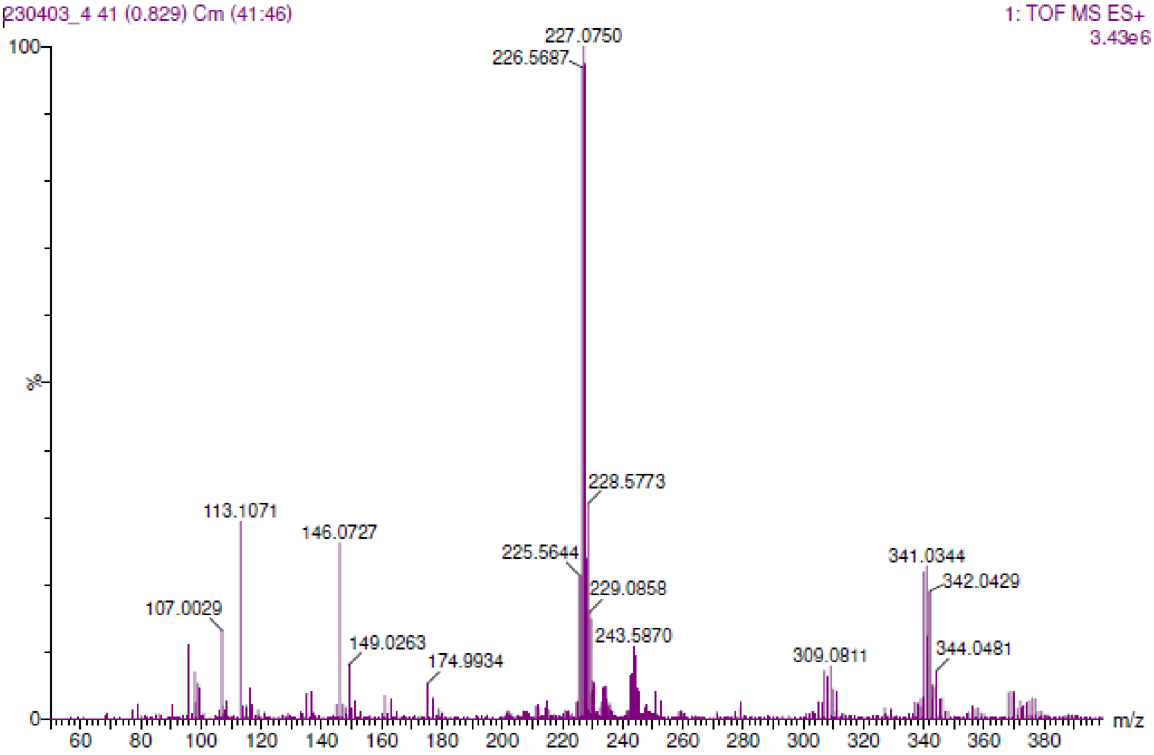

Figure S46. ESIMS spectrum of [Pt(ImPy)(*RR*DACH)]$^{+2}$ measured on a Waters TQ-MS triple quadrupole mass spectrometer. Sample solutions were made up to 0.3 mM in H_2O: MeOH (90:10) and run at 0.1 mL/min.

[Pt(BImPy)(*SS*DACH)]$^{2+}$

Elemental Composition Report **Page 1**

Single Mass Analysis
Tolerance = 20.0 PPM / DBE: min = -1.5, max = 50.0
Element prediction: Off
Number of isotope peaks used for i-FIT = 9

Monoisotopic Mass, Even Electron Ions
72 formula(e) evaluated with 2 results within limits (all results (up to 1000) for each mass)
Elements Used:
C: 0-20 H: 0-30 N: 0-6 Na: 0-1 Br: 0-1 Pt: 1-1

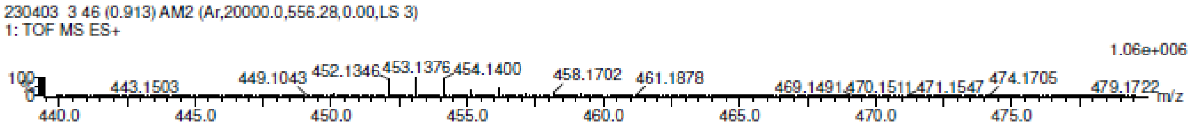

Minimum:				-1.5				
Maximum:		20.0	20.0	50.0				
Mass	Calc. Mass	mDa	PPM	DBE	i-FIT	Norm	Conf(%)	Formula
453.1376	453.1366	1.0	2.2	8.5	1622.0	0.553	57.54	C14 H20 N5 Pt
	453.1342	3.4	7.5	5.5	1622.3	0.857	42.46	C12 H21 N5 Na Pt

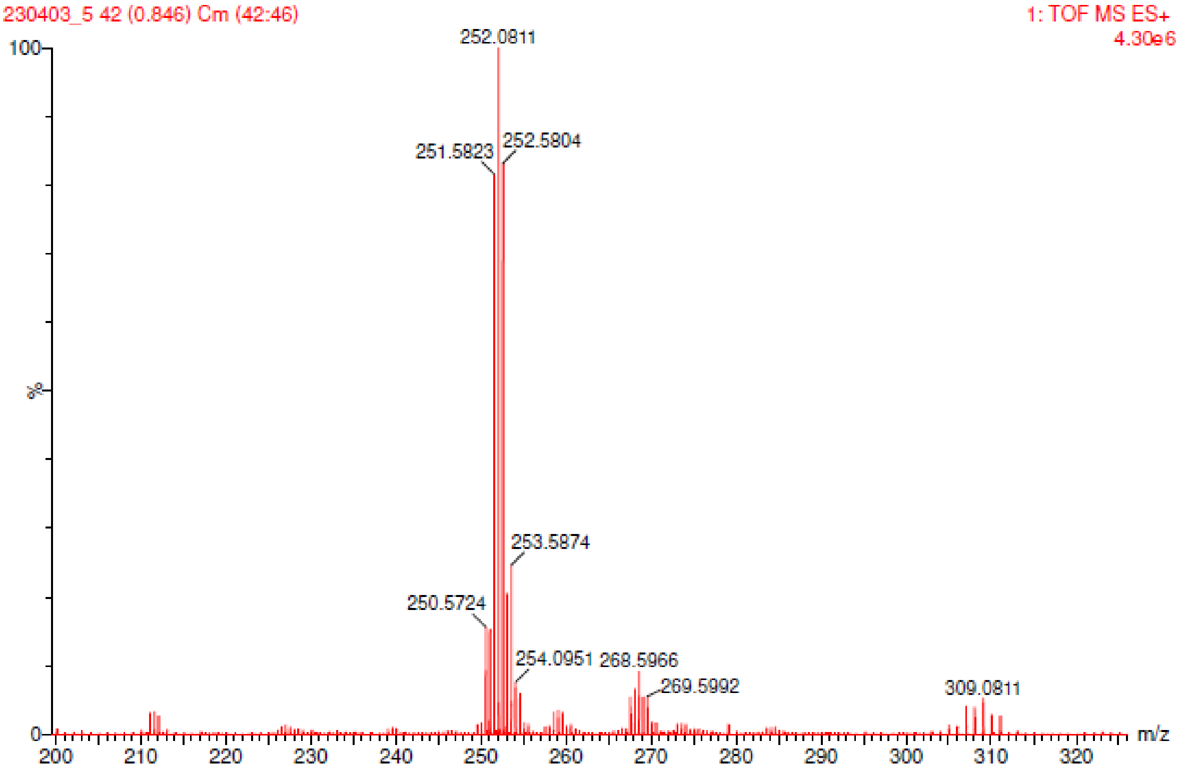

Figure S47. ESIMS spectrum of [Pt(BImPy)(*SS*DACH)]$^{+2}$ measured on a Waters TQ-MS triple quadrupole mass spectrometer. Sample solutions were made up to 0.3 mM in H_2O: MeOH (90:10) and run at 0.1 mL/min.

[Pt(BImPy)(*RR*DACH)]$^{2+}$

Elemental Composition Report **Page 1**

Multiple Mass Analysis: 3 mass(es) processed
Tolerance = 20.0 PPM / DBE: min = -1.5, max = 50.0
Element prediction: Off
Number of isotope peaks used for i-FIT = 9

Monoisotopic Mass, Even Electron Ions
178 formula(e) evaluated with 6 results within limits (all results (up to 1000) for each mass)
Elements Used:
C: 0-20 H: 0-30 N: 0-6 Na: 0-1 Br: 0-1 Pt: 1-1

230403 6 17 (0.364) AM2 (Ar,20000.0,0.00,0.00); Cm (16:27)
1: TOF MS ES+

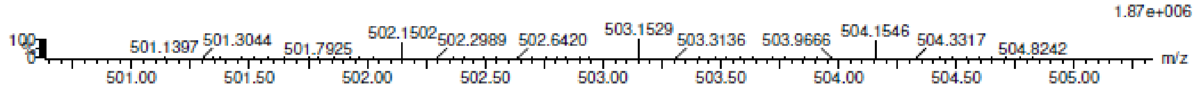

Mass	RA	Calc. Mass	mDa	PPM	DBE	i-FIT	Norm	Conf(%)	Formula
Minimum:	80.00				-1.5				
Maximum:	100.00		20.0	20.0	50.0				
502.1502	82.05	502.1546	-4.4	-8.8	8.5	611.4	0.585	55.73	C17 H24 N4 Na Pt
		502.1570	-6.8	-13.5	11.5	611.6	0.815	44.27	C19 H23 N4 Pt
503.1529	100.00	503.1523	0.6	1.2	11.5	403.7	0.537	58.47	C18 H22 N5 Pt
		503.1499	3.0	6.0	8.5	404.1	0.879	41.53	C16 H23 N5 Na Pt
504.1546	90.01	504.1475	7.1	14.1	11.5	200.0	0.687	50.31	C17 H21 N6 Pt
		504.1451	9.5	18.8	8.5	200.0	0.699	49.69	C15 H22 N6 Na Pt

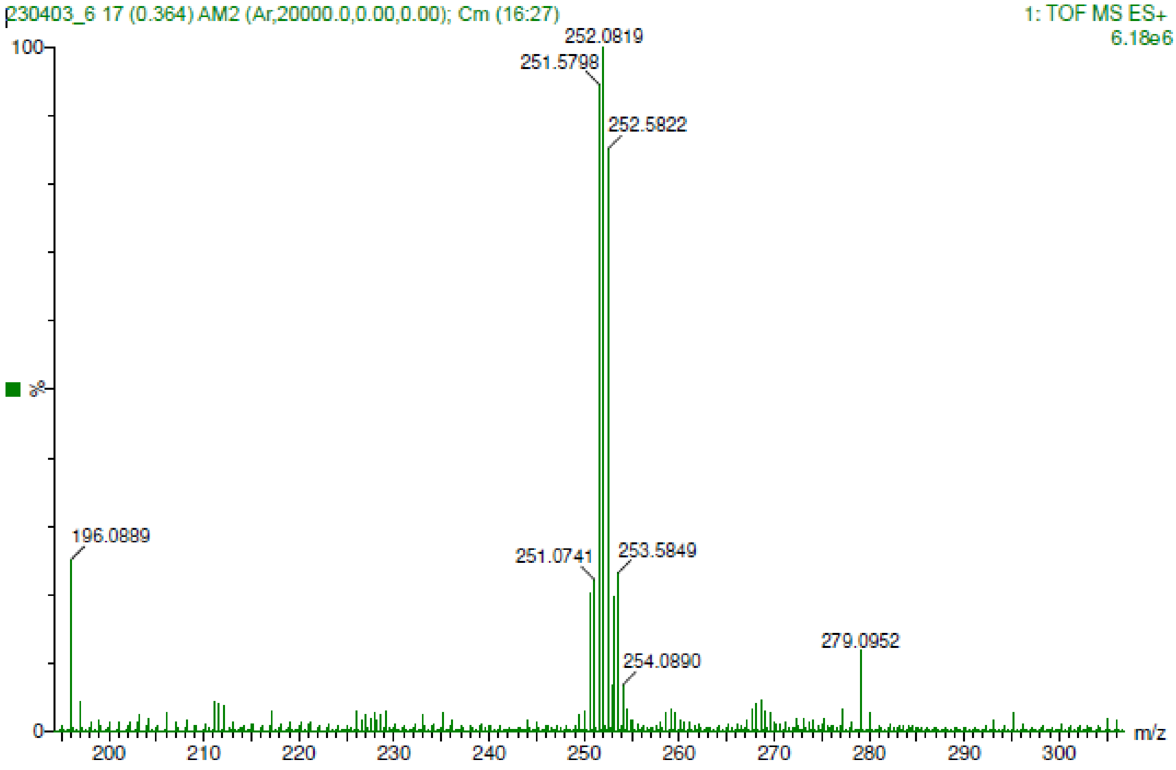

Figure S48. ESIMS spectrum of [Pt(BImPy)(*RR*DACH)]$^{+2}$ measured on a Waters TQ-MS triple quadrupole mass spectrometer. Sample solutions were made up to 0.3 mM in H_2O: MeOH (90:10) and run at 0.1 mL/min.

Lipophilicity

Figure S49 illustrates the linear plots of logK' against % ACN including the resulting equation. These experiments were undertaken on an Agilent Technologies 1260 Infinity machine equipped with a Phenomenex Onyx™ Monolithic C18 reverse phase column (100 × 4.6 mm, 130 Å). The mobile phase comprised of 0.06% TFA in water (solvent A) and 0.06% TFA in ACN.H_2O (90 . 10, solvent B). The dead time was determined using potassium iodide as an external dead volume marker.

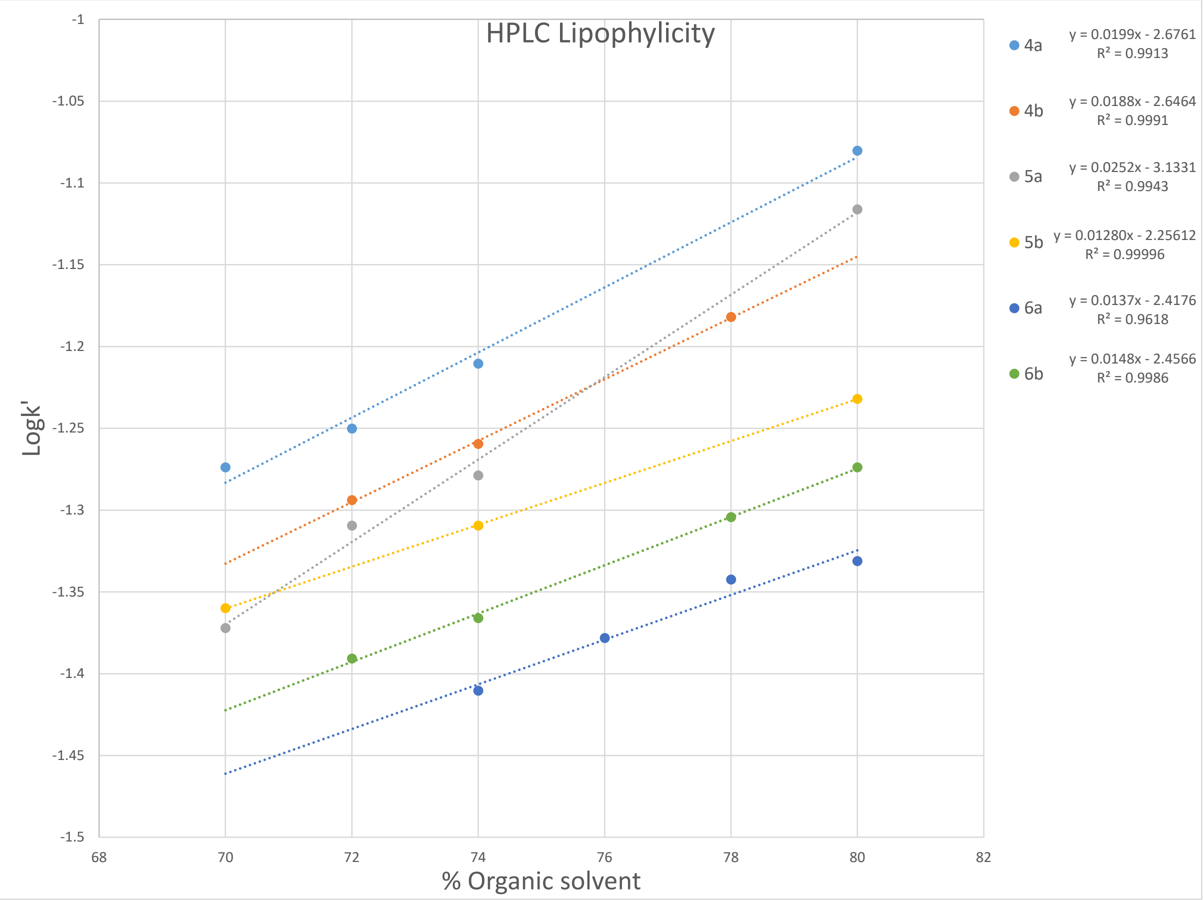

Figure S49. LogK' plotted against % ACN in the mobile phase.

References

1. Klose, M.H.M.; Theiner, S.; Varbanov, H.P.; Hoefer, D.; Pichler, V.; Galanski, M.; Meier-Menches, S.M.; Keppler, B.K. Development and Validation of Liquid Chromatography-Based Methods to Assess the Lipophilicity of Cytotoxic Platinum(IV) Complexes. *Inorganics* **2018**, *6*, 130 doi:10.3390/inorganics6040130.
2. Reithofer, M.R.; Bytzek, A.K.; Valiahdi, S.M.; Kowol, C.R.; Groessl, M.; Hartinger, C.G.; Jakupec, M.A.; Galanski, M.; Keppler, B.K. Tuning of Lipophilicity and Cytotoxic Potency by Structural Variation of Anticancer Platinum(IV) Complexes. *J. Inorg. Biochem.* **2011**, *105*, 46–51, doi:10.1016/j.jinorgbio.2010.09.006.
3. Valkó, K. Application of High-Performance Liquid Chromatography Based Measurements of Lipophilicity to Model Biological Distribution. *J. Chromatogr. A* **2004**, *1037*, 299–310, doi:10.1016/j.chroma.2003.10.084.
